# Structure of the PCNA unloader Elg1-RFC

**DOI:** 10.1126/sciadv.adl1739

**Published:** 2024-03-01

**Authors:** Fengwei Zheng, Nina Y. Yao, Roxana E. Georgescu, Huilin Li, Michael E. O’Donnell

**Affiliations:** ^1^ Department of Structural Biology, Van Andel Institute, Grand Rapids, MI, USA.; ^2^ DNA Replication Laboratory and Howard Hughes Medical Institute, The Rockefeller University, NY, New York, USA.

## Abstract

During DNA replication, the proliferating cell nuclear antigen (PCNA) clamps are loaded onto primed sites for each Okazaki fragment synthesis by the AAA^+^ heteropentamer replication factor C (RFC). PCNA encircling duplex DNA is quite stable and is removed from DNA by the dedicated clamp unloader Elg1-RFC. Here, we show the cryo-EM structure of Elg1-RFC in various states with PCNA. The structures reveal essential features of Elg1-RFC that explain how it is dedicated to PCNA unloading. Specifically, Elg1 contains two external loops that block opening of the Elg1-RFC complex for DNA binding, and an “Elg1 plug” domain that fills the central DNA binding chamber, thereby reinforcing the exclusive PCNA unloading activity of Elg1-RFC. Elg1-RFC was capable of unloading PCNA using non-hydrolyzable AMP-PNP. Both RFC and Elg1-RFC could remove PCNA from covalently closed circular DNA, indicating that PCNA unloading occurs by a mechanism that is distinct from PCNA loading. Implications for the PCNA unloading mechanism are discussed.

## Teaser

The structure of the yeast Elg1-RFC is compatible with unloading PCNA from DNA but not with loading PCNA onto DNA.

## INTRODUCTION

Faithful passage of parental genome information into progeny cells is a critical life process for all cell types (*1*). Genome DNA replication and DNA damage repair are central to this paramount mission of maintaining genome integrity, and failure to do so can lead to apoptosis or cancer (*2*).

Numerous proteins function together to replicate the genome and to repair damaged DNA (*1*, *2*). One of the pivotal factors that function in replication and repair is the DNA sliding clamp, a ring-shaped oligomer that encircles DNA and is essential in all three domains of life (*3*–*5*). In eukaryotes, the sliding clamp is the proliferating cell nuclear antigen (PCNA) homo-trimeric ring (*5*) that is typically loaded onto 3′ primed sites by the clamp loader replication factor C (RFC) (*6*), a heteropentamer that belongs to the AAA^+^ adenosine triphosphatase family. Upon binding adenosine 5′-triphosphate (ATP), RFC can open PCNA into a “lock washer” shape and load it onto a 3′ single-strand/double-strand DNA (ss/dsDNA) junction (*7*–*9*). Then, PCNA can recruit various factors, such as the replicative polymerases (Pols), Pols delta, and epsilon, and hold them to DNA while sliding along behind them to provide them with processivity during DNA synthesis as originally shown for *Escherichia coli* replication (*3*, *10*–*12*) or to recruit trans-lesion synthesis (TLS) Pols to traverse sites of damaged DNA when needed (*13*).

Unlike bacteria and archaea, eukaryotes have evolved three other RFC-like complexes: *Saccharomyces cerevisiae* (S.c.) radiation protein 24 (Rad24)–RFC (human Rad17-RFC), chromosome transmission fidelity protein 18 (Ctf18)–RFC, and S.c. enhanced levels of genome instability 1 (Elg1)–RFC (human ATAD5-RFC) (*14*, *15*). The three alternative RFC-like complexes are formed by replacement of the large subunit, Rfc1 of RFC, with Rad24, Ctf18, or Elg1 (*16*). Unlike the other RFC-like complexes, Rad24-RFC loads a unique DNA sliding clamp heterotrimer ring composed of 9-1-1 (human Rad9-Hus1-Rad1) onto a 5′ ss/ds junction (*17*, *18*), mainly involved in the DNA damage cell cycle checkpoint pathway to arrest cells until DNA is repaired (*19*, *20*). Later studies also showed that 9-1-1 can be loaded onto the 3′ end of DNA with a medium sized gap, indicating a role in DNA repair by associating with TLS Pols (*21*). Ctf18-RFC is also a PCNA loader, suggested to function for the leading strand (*22*, *23*), and also plays a role in sister chromatid cohesion establishment (*24*).

**Fig. 1.**
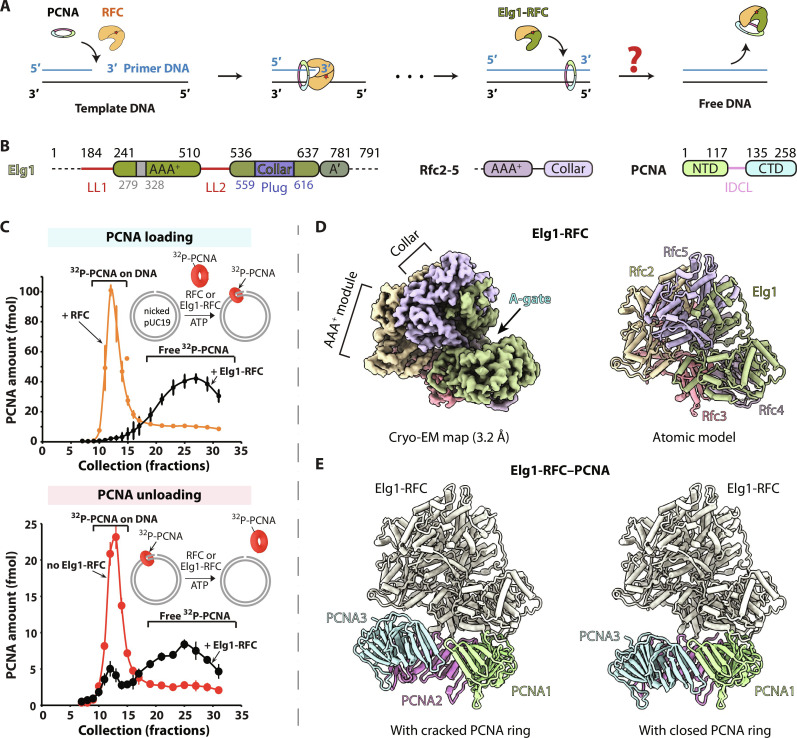
PCNA clamp unloading by the Elg1-RFC unloader. (**A**) Comparison of PCNA loading by RFC and PCNA unloading by Elg1-RFC. How Elg1-RFC unloads PCNA from dsDNA (i.e., after DNA replication) remains unknown. (**B**) Domain architecture of Elg1-RFC, Rfc2-5, and the PCNA clamp. The Rfc2-5 subunits each contain a AAA^+^ module and a C-terminal “collar” domain. Elg1 also contains these elements but has an insertion in the collar domain that forms the plug inside the central chamber and has two large locking loops, LL1 and LL2 (purple), that prevent conformation changes necessary for PCNA opening and DNA binding. Each PCNA monomer has two structurally similar globular domains [N-terminal domain (NTD) and C-terminal domain (CTD)] that give the PCNA trimer ring a sixfold pseudo-symmetry; they are linked by the inter-domain connecting loop (IDCL). (**C**) Elg1-RFC is competent for PCNA unloading but not loading. RFC is adopted as a positive control for the ^32^P-PCNA loading assay, and a plasmid DNA pUC19 was used to detect the loading and unloading activities of Elg1-RFC for the ^32^P-labeled PCNA clamp. Reactions were gel-filtered to separate the large ^32^P-PCNA-DNA complex from the smaller “free” ^32^P-PCNA (see Materials and Methods for details). Three independent experiments were performed for both the top and bottom experiments, and data points are presented as mean (filled circles) ± SD (error bars). (**D**) The 3.2-Å resolution EM map and atomic model of Elg1-RFC colored by subunits. The position of the A-gate (between the Elg1 AAA^+^ module and A′ domain) is also labeled. The locking loop density is invisible at the high surface rendering threshold used here. (**E**) The atomic models of Elg1-RFC complexed with a cracked or a closed PCNA ring. For clarity, the Elg1-RFC pentamer is colored ivory.

Elg1-RFC is the latest RFC-like complex discovered (Fig. 1, A and B) (*16*, *25*, *26*). Like the other RFC-like complexes, it is not essential for viability, but deletion of *Elg*1 leads to severe growth defects, including increased spontaneous DNA damage, elevated homologous recombination, chromosome loss, and gross chromosome rearrangements (*16*, *27*–*29*). Loss of Elg1 also enhances cell sensitivity to DNA damage reagents and leads to elongated telomeres (*30*–*33*). Moreover, the mammalian ortholog of Elg1 (ATAD5) is a tumor suppressor in mice and is associated with cancer in humans (*34*). Human ATAD5 is also reported to function in the Fanconi anemia pathway (*35*, *36*).

In sum, the important role of Elg1 in unloading PCNA and in maintaining genome stability and integrity has been established by numerous genetic and cell-based studies (*16*, *25*, *26*, *29*, *32*). However, the structure of Elg1-RFC and why it does not appear to load PCNA onto DNA, as supported in results of the current report, remain unknown. Elg1-RFC is reported to unload PCNA from chromatin in vitro using extracts (*37*) and pure proteins, including ubiquitinated and SUMOylated versions of PCNA (*37*–*42*). However, it is also important to note that all clamp loaders have also been demonstrated to be capable of unloading PCNA from DNA in vitro (*24*, *41*, *43*), and, therefore, it is not clear whether RFC, Ctf18-RFC, or Elg1-RFC is the true PCNA unloader (*15*, *44*). While the loading of replication clamps onto DNA is congruent with the structures of the clamp loaders, why Elg1-RFC seems devoted to PCNA unloading is still enigmatic (Fig. 1A). By leveraging state-of-the-art cryo–electron microscopy (cryo-EM), we find that the architecture of Elg1-RFC is specifically suited to the unloading of PCNA. This report documents Elg1-RFC alone and Elg1-RFC bound to a closed PCNA clamp and to a cracked PCNA clamp at 3.2-, 3.3-, and 3.7-Å resolution, respectively. The structures support the fact that Elg1-RFC can only unload PCNA, distinguishing it from RFC and the other RFC-like complexes that can load clamps onto DNA. Furthermore, our biochemical experiments reveal that nucleotide binding, not hydrolysis, is sufficient for Elg1-RFC–mediated PCNA unloading. We observe that RFC (and Elg1-RFC) can remove PCNA from a covalently closed circular DNA, implying the PCNA unloading process is not on the same pathway as PCNA loading, which requires a flexible ss/dsDNA junction.

## RESULTS

### Elg1-RFC unloading of PCNA and inability to load PCNA

Early in vitro studies using pure yeast proteins did not observe unloading by Elg1-RFC (*24*), although later studies using pure proteins observed unloading by Elg1-RFC (*41*, *42*). Thus, we first characterized the loading and unloading activity of the Elg1-RFC preparation used in the current study. We expressed Elg1-RFC in yeast and first tested its activity for PCNA clamp loading/unloading using ^32^P-labeled PCNA and a nicked pUC19 plasmid as a DNA substrate (Fig. 1C and fig. S1A). For a control, we used the canonical RFC clamp loader. For loading, the reactions were applied to a gel filtration column to resolve the large ^32^P-PCNA-DNA complex from free PCNA, followed by quantification of the ^32^P-PCNA clamp in each fraction (see Materials and Methods). RFC efficiently loaded ^32^P-PCNA onto DNA, but the Elg1-RFC did not load ^32^P-PCNA onto DNA, as indicated by the absence of ^32^P-PCNA comigration with the large DNA in fractions 10 to 15 of the gel filtration profile, and, instead, the ^32^P-PCNA eluted in fractions 20 to 30 containing the free ^32^P-PCNA. Recent studies showed that the loading of ^32^P-PCNA or ^32^P- 9-1-1 clamp onto a gapped DNA is more efficient than loading onto DNA with a single 3′ or 5′ end (*8*, *21*). Therefore, we engineered a 20–nucleotide (nt) gapped pUC19 plasmid for these assays but still observed no loading over a wide concentration range (15.4 to 121.2 nM) by Elg1-RFC (fig. S1B).

To be sure that our Elg1-RFC preparation was active in PCNA unloading, ^32^P-PCNA was first loaded onto the DNA using pure RFC, and, then, the reaction was gel-filtered to remove RFC and free ^32^P-PCNA. We then treated the purified ^32^P-PCNA-DNA with either Elg1-RFC or a buffer control. Using Elg1-RFC, we observed a peak shift from fractions 10 to 15 (^32^P-PCNA loaded) to 20 to 30 (^32^P-PCNA unloaded), whereas the buffer control did not unload PCNA (Fig. 1C). Elg1-RFC can also unload PCNA from the pUC19 plasmid containing a 20-nt gap (fig. S1C). This unloading activity is consistent with previous studies of Elg1-RFC (*37*, *39*, *42*). As a control, we incubated Elg1-RFC (75 nM) with supercoiled pUC19 plasmid and observed no nuclease cleavage of DNA (fig. S1D), demonstrating that the observed ^32^P-PCNA unloading is due to Elg1-RFC and not PCNA sliding off pUC19 DNA linearized by a contaminating nuclease.

### Cryo-EM structures of the PCNA clamp unloading complex

**Fig. 2.**
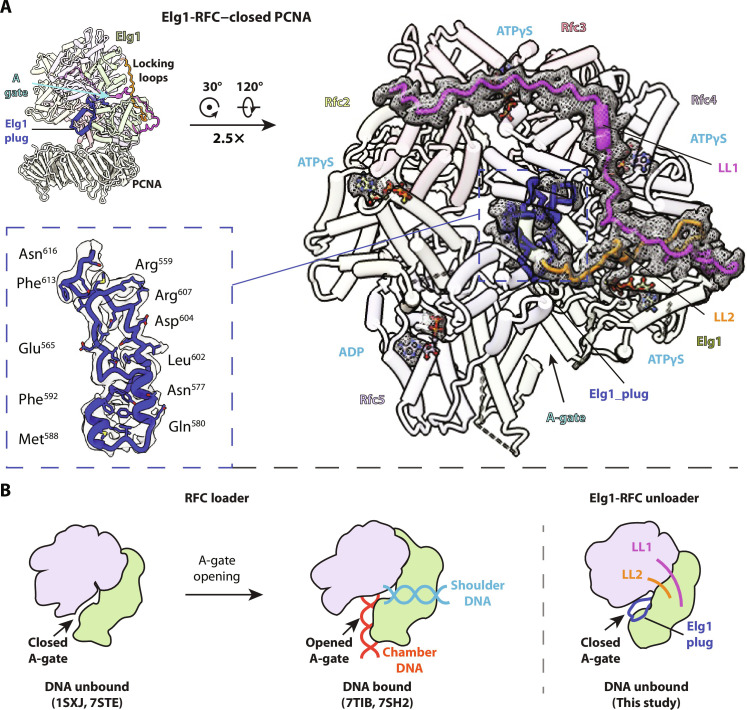
The A-gate in Elg1-RFC is locked in the closed state by two Elg1 loops. (**A**) The front (left) and top (top right, 2.5× enlarged) view of Elg1-RFC (PCNA unloader). Compared with Rfc1 subunit, the Elg1 has an additional loop LL1 to enhance the locking of the A-gate in the closed state. The Elg1 LL2 is equivalent to the Rfc1 alternative linker that can partially unfold to allow the opening of the A-gate (*8*, *9*, *79*). The local density of five bound nucleotides and LL1 and LL2 is shown as meshes, and the Elg1 plug is boxed by a blue dashed line and enlarged in middle left. The PCNA ring is closed and is omitted in the top view for clarity. (**B**) A diagram showing that the A-gate in the RFC clamp loader can be opened to bind both shoulder and central chamber DNA to accommodate the loading of PCNA clamp, while infeasible in the PCNA unloader, Elg1-RFC, due to the Elg1 locking loops and Elg1 plug.

We next set out to understand the structural basis that explains why Elg1-RFC lacks PCNA loading activity by cryo-EM. Thus, we mixed PCNA, Elg1-RFC, and a 5′-tailed DNA substrate in the presence of 1 mM of the slowly hydrolyzable ATP analog (ATPγS) and prepared cryo-EM grids similar to reported previously for the assembly of the ternary complexes of RFC-PCNA-DNA and Rad24-RFC–9-1-1–DNA (also see Materials and Methods) (*8*, *10*). Class-averaged images of cryo-EM raw particles showed the successful reconstitution of the binary Elg1-RFC–PCNA, but without any DNA density (fig. S1, E and F). The absence of DNA in all observed Elg1-RFC–PCNA complexes is consistent with Elg1-RFC acting as a PCNA unloader, rather than as a PCNA loader. After several rounds of refinement, we obtained three EM maps: one map of Elg1-RFC alone, one map of Elg1-RFC bound to a closed PCNA ring, and one map of Elg1-RFC bound to PCNA with a cracked interface, at overall resolutions of 3.2, 3.3, and 3.7 Å, respectively (Fig. 1, D and E; figs. S2 and S3; and table S1). In all three Elg1-RFC structures, we found five bound nucleotides: four ATPγS in Rfc2, Rfc3, Rfc4, and Elg1, and one ADP in Rfc5 (Fig. 2A, top right). This nucleotide binding pattern in Elg1-RFC is comparable to all structurally characterized clamp loaders from T4 phage to *E. coli*, yeast, and human (*7*–*9*, *45*–*47*) and is also similar to the 9-1-1 clamp loader Rad24-RFC (*17*, *18*).

The Elg1 structures reveal two remarkable features that stand in contrast to clamp loaders (Fig. 2A). In clamp loaders, the binding of nucleotide (and clamp and DNA) to RFC or Rad24-RFC enable large conformational changes that create an opening between the A and A′ domains (i.e., the “A-gate”) of either Rfc1 or Rad24, needed to enable DNA passage into the central DNA binding chamber of the circular clamp loader pentamer (*7*–*9*). Furthermore, these conformational changes in RFC and Rad24-RFC that enable DNA binding are the result of a large ATP-binding induced rotation between the AAA^+^ domains and the collar domains, which open the A-gate and they also produce a second DNA site for 5′ ss/dsDNA binding on the outside “shoulder” of the Rfc1 or Rad24 subunit (*7*–*9*). However, Elg1 has evolved two loops just above the A-gate that prevent the A-gate from opening (*7*–*9*). The two loops in Elg1 lock the A-gate shut, creating a structure that cannot undergo the extensive rotational changes required for binding DNA in the central chamber, and also block DNA from entering the external 5′ ss/dsDNA site. Hereafter, we refer to these two loops as the “locking loops” (LL1 and LL2). Second, there is a unique plug domain inside the central DNA binding chamber of Elg1-RFC that normally accommodates dsDNA in the PCNA loader complexes. The plug domain is formed by an interesting rearrangement of the collar domain of Elg1 and will be described in more detail below. These structural features, the loops and plug, would appear to prevent Elg1-RFC from binding DNA inside the central chamber or outside in the 5′ ss/ds shoulder site. We presume these features define the exclusivity of Elg1-RFC to PCNA unloading and not PCNA loading (Fig. 2B). While these large-scale features are obvious blocks to clamp loading, we did not attempt mutagenesis given that single site substitutions are unlikely to prevent activity and that larger mutagenesis may result in improper folding. In this connection, it should be noted that we have previously shown that even the subcomplexes Rfc2-4 and Rfc2 and Rfc5 can unload PCNA when present at elevated concentration that could confuse in vitro studies of PCNA unloading by mutant Elg1-RFC complexes because they contain Rfc2-4 (*43*).

Because clamp unloading starts with PCNA encircling dsDNA (*39*), we propose that the three structures that we have determined represent (i) Elg1-RFC alone before it encounters a PCNA, (ii) Elg1-RFC that has taken the PCNA off DNA with the clamp being incompletely closed (i.e., cracked PCNA), and (iii) Elg1-RFC bound to the closed PCNA after PCNA is taken off DNA (Elg1-RFC–closed PCNA), respectively (Fig. 1, D and E, and movie S1). The shared Elg1-RFC among the three structures is highly similar with a root mean square deviation between main chain C_α_ atoms in the range of 0.5 to 0.6 Å. In the cracked PCNA ring, PCNA protomers 2 and 3 are slightly uplifted toward the unloader, disrupting the β sheet interface between a PCNA interface (Fig. 1E). Clamp loader disruption of a PCNA interface is also observed in the RFC-CNA-DNA complexes, but, in those cases, the PCNA forms a wide-open ring, guided by the large RFC conformational changes, and is a presumed intermediate in clamp loading rather than unloading (*7*–*9*, *45*).

### The Elg1 locking loops LL1 and LL2

**Fig. 3.**
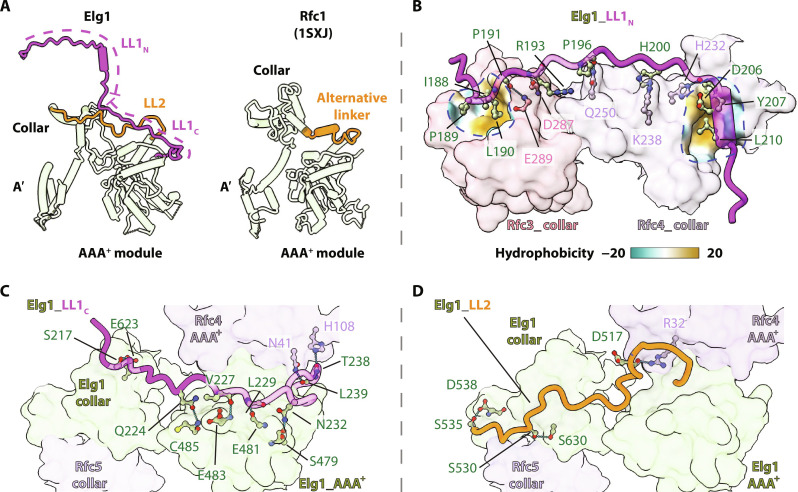
The stabilization of the two Elg1 locking loops. (**A**) Left: The two linkers of Elg1 are shown as LL1_N_ and LL1_C_ (magenta) and LL2 (orange), compared with the alternative linker of Rfc1 (right, orange) that corresponds to the LL2 linker of Elg1. (**B** to **D**) The stabilization of Elg1 LL1_N_ (B), LL1_C_ (C), and LL2 (D). (B) Elg1 LL1_N_ (in cartoons) wraps around the collar (in semitransparent surface view, also see Fig. 2A) and is mainly stabilized by two hydrophobic pockets that are demarcated by blue dashed circles and overlaid as hydrophobicity surface plots. (C and D) The LL1_C_ (C) and LL2 (D) (i.e., positioned above the A-gate) are mainly stabilized by hydrophilic interactions. Key residues involved are shown as sticks, and H-bonds as cyan dashed lines.

The Elg1 locking loops LLI and LL2 are located right above the A-gate and are unique to Elg1 and absent in RFC and Rad24-RFC (Fig. 2A and Fig. S4). The first loop (LL1) is 57-residue long between Arg^184^ and Thr^240^, preceding the AAA^+^ domain and emanating from between the collar domains of Rfc2 and Rfc3 and wraps around the collar domains of Rfc3 and Rfc4, whereby the C-terminal half (LL1_C_) cross-links the A-gate (Fig. 2A, top right). The LL2 loop is 25-residue long (Cys^511^ to Ser^535^) and corresponds to the “alternative linker” loop in Rfc1 of RFC (Figs. 2A and 3A) (*7*–*9*). Together, the Elg1-RFC A-gate is locked by the LL1 and LL2 loops into a closed and static state, which is unable to open to bind DNA (Fig. 2B). A deletion analysis of Elg1 shows residual unloading activity even when the N-terminal 215 residues are omitted (*42*). This deletion would include some of the LL1, suggesting that LL2 alone may be sufficient to lock the Elg1-RFC closed.

The numerous details of the LL1 and LL2 loop interactions with Elg1-RFC are shown in Fig. 3 (B to D). Elg1 LL1_N_ is stabilized by a mixture of hydrophobic and hydrophilic interactions (Fig. 3B). There are two hydrophobic pockets on the surface of the Elg1-RFC unloader: one at the side of the Rfc3 collar domain that is involved in interaction with Ile^188^, Pro^189^, and Leu^190^ of the LL1_N_; and the other between the AAA^+^ domain and collar domain of Rfc4 that interacts with the LL1_N_ Asp^206^ and Tyr^207^ and Leu^210^ from its only helix α0 (Fig. 3B and fig. S4). In addition, there are several H-bonds between the LL1_N_ and the Elg1-RFC collar tier, including the LL1 Pro^191^ and Arg^193^ with Rfc3 Glu^289^ and Asp^287^, respectively, among their main-chain atoms, Pro^196^ main-chain oxygen, His^200^ and Tyr^207^ with Rfc4 Gln^250^, Lys^238^, and His^232^, respectively (Fig. 3B). The LL1_C_ and LL2 are mainly stabilized by hydrophilic interactions (Fig. 3, C and D). The LL1_C_ Ser^217^, Gln^224^, Val^227^, Leu^229^, and Asn^232^ form H-bonds with the Elg1 AAA^+^ domain Glu^623^, Cys^485^, Glu^483^, Glu^481^, and Ser^479^, respectively, and the LL1_C_ Thr^238^ and Leu^239^ form H-bonds with the Rfc4 His^108^ and Asn^41^, respectively (Fig. 3C); the LL2 Asp^517^, Ser^530^, and Ser^535^ form H-bonds with the Rfc4 Arg^32^ and the Elg1 collar domain Ser^630^ and Asp^538^, respectively (Fig. 3D).

In contrast, the RFC and the 9-1-1 DNA damage checkpoint clamp loaders also contain a linker loop, but it is short and sits below the 5′ ss/ds external DNA shoulder binding site, thereby enabling A-gate opening and external site DNA binding (Fig. 3A, right) (*7*–*9*, *17*, *18*, *48*). The Elg1-RFC LL1 and LL2 linkers are stretched and stabilized by extensive interactions and cannot further extend; therefore, they lock the A-gate in place and prevent DNA insertion into the central chamber of Elg1-RFC, as well as blocking access to any potential external DNA site (Fig. 2B). This structural feature precludes the Elg1-RFC to function as a PCNA loader which requires the ability to bind DNA in the central chamber as described further below. Therefore, our structure explains why Elg1-RFC is exclusively a PCNA unloader (Fig. 2, A and B).

### The Elg1 plug

**Fig. 4.**
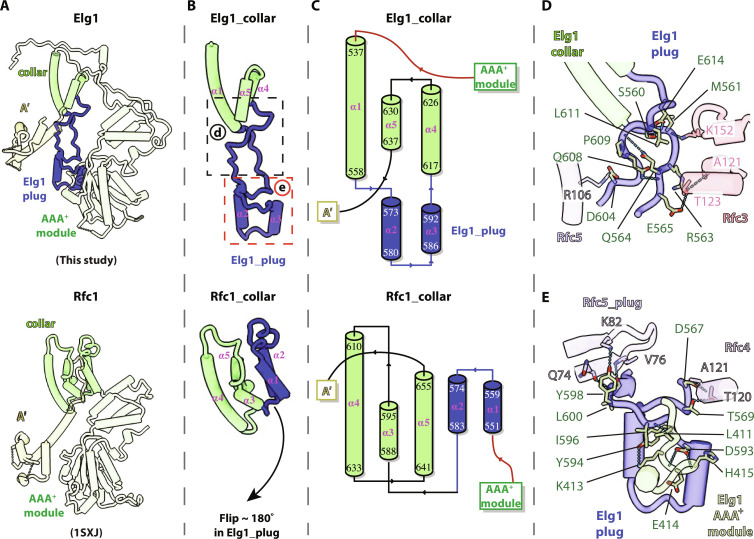
The Elg1 collar domain has evolved a plug that occupies the central chamber of the Elg1-RFC unloader. (**A**) Comparison of full length Elg1 (top) with Rfc1 (bottom). Elg1 and Rfc1 are colored by domain. (**B**) The five α helices in the collar domain of Elg1 (top) are compared with the Rfc1 collar domain (bottom); two of these α helices are flipped ~180° downward to form the Elg1 plug that occupies the Elg1-RFC central chamber and helps exclude DNA from the central chamber. (**C**) Topological comparison of the collar domains of Elg1 (top) versus Rfc1 (bottom) reveals that the Elg1 collar domains have rearranged to form the plug that inserts between the AAA^+^ modules and occludes DNA from the central DNA binding chamber (also see movie S2). (**D** and **E**) The two boxed regions of Egl1 in (B) are enlarged and placed in context of the Elg1-RFC pentamer. The AAA^+^ domains of Rfc3, Rfc4, and Rfc5 (but not Rfc2) contact and thus stabilize the Elg1 plug. The H-bonds, including intra-molecular ones, are shown as dashed lines in both panels, and the Elg1 plug forms a short parallel β sheet with the Rfc5 plug [i.e., top left of (E)]. See section "The Elg1 plug" for details.

An additional feature of the Elg1-RFC that defines it as a clamp unloader is the Elg1 plug that almost entirely fills up the central DNA binding chamber (Figs. 2A and 4A and fig. S5), making it incompatible with DNA binding. The Elg1 plug is distinct from and is in addition to the well-established Rfc5 plug that interacts with the major groove of the dsDNA inside the central chamber of a clamp loader (*49*). DNA binding inside the central chamber, formed by all five subunits and accessed by widening the A-gate between the A and A′ domains of Rfc1 (or Rad24-RFC), is a required feature of both RFC (the PCNA loader) and Rad24-RFC (the 9-1-1 loader). Elg1 shares with Rfc1 (and Rad24) a similar N-terminal AAA^+^ module and C-terminal A′ domain (Fig. 4A), but the collar domain of Elg1 is markedly different from Rfc1 (Fig. 4B). The Rfc1 collar domain contains five α helices, but two of the five α helices in the Elg1 collar domain have descended to form the Elg1 plug, with only three α helices remaining at the top collar tier. The collar domains of Elg1 and Rfc1 have a notably different topology (Fig. 4C). The three Elg1 α helices at the collar tier (α1, α4, and α5) are structurally equivalent to α4, α5, and α3 in the Rfc1 collar domain, and the descended α helices α2 and α3 (the plug) are structurally equivalent to α1 and α2 of the Rfc1 collar domain, although they are flipped upside down in the Elg1 structure. Therefore, evolution of Rfc1 to Elg1 is not a simple modification of primary sequence but, instead, involves gross rearrangement of secondary structure elements in the collar domain (Fig. 4, B and C, and movie S2). In Rfc1, the short linker connecting the collar domain to the AAA^+^ module is located at the bottom of the 5′ DNA binding groove on the external shoulder site and is compatible with DNA binding. In contrast, both the LL1 and LL2 loops of Elg1 are positioned above the groove between the collar tier and the AAA^+^ module, which would block DNA from attaining a close approach to the location of an external shoulder site (Fig. 4, A and C). The electrostatic surface of Elg1 is dominated by acidic residues and is not as basic as in Rfc1 of the RFC loader (fig. S6), thereby further excluding its use as a DNA binding site.

The Elg1 plug interacts with and is extensively stabilized by all Elg1-RFC subunits except for Rfc2 (Fig. 4, D and E). In the top region, the Elg1 plug is stabilized by Rfc3 and Rfc5 and several intra-Elg1 plug H-bonds. The Elg1 plug Met^561^, Arg^563^, Glu^565^, and Asp^604^ form H-bonds with the Rfc3 Lys^152^, Ala^121^, and Thr^123^ and with the Rfc5 Arg^106^, respectively (Fig. 4D). Within the Elg1 plug, Gln_564_ forms two H-bonds with Gln^608^ and another H-bond with Pro^609^, and Glu^614^ forms three H-bonds with Ser^560^, Met^561^, and Leu^611^, respectively (Fig. 4D). In the middle region, the Elg1 plug Asp^567^ and Thr^569^ H-bond with the Rfc4 Ala^121^ and Thr^120^, respectively, and the Elg1 plug Tyr^598^ form a H-bond with the Rfc5 plug Lys^82^. Further, the Elg1 plug Leu^600^ and His^601^ form a short parallel β sheet interaction with the Rfc5 plug (Fig. 4E and fig. S5A). In the bottom region, the Elg1 plug Asp^593^, Tyr^594^, and Ile^596^ H-bond with the Elg1 AAA^+^ module Glu^414^, His^415^, Lys^413^, and Leu^411^ (Fig. 4E). Given the extensive interactions, the Elg1 plug is likely immobile during the PCNA unloading process.

### The Elg1 noncanonical PIP motif for PCNA binding

**Fig. 5.**
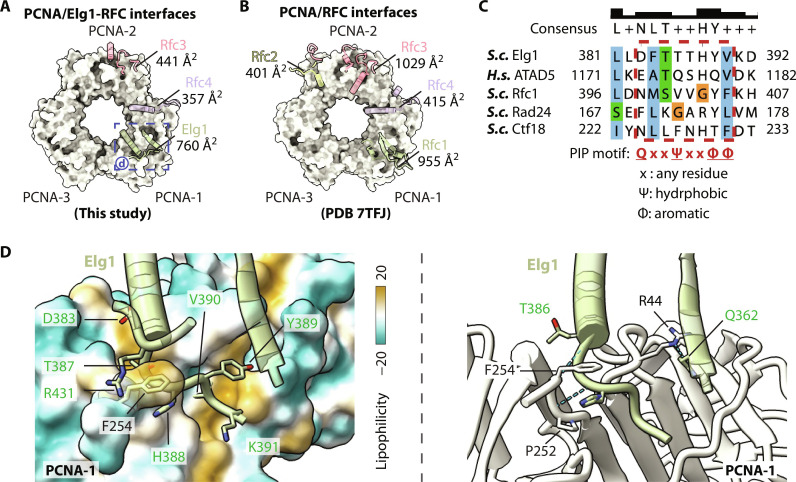
Comparison of the interfaces between PCNA and Elg1-RFC and between PCNA and RFC. (**A** and **B**) The binding interfaces of Elg1-RFC (A) and loader RFC (B) on PCNA clamp. The PCNA trimer is shown as surface. For clarity, only the Elg1/RFC regions involved in PCNA binding are shown as cartoons. The interacting subunits and the buried solvent-accessible surface area are labeled. (**C**) Structural-based sequence alignment suggests a noncanonical PIP motif in Elg1. The structures used are *Saccharomyces cerevisiae* (***S.c.***) Elg1-RFC–closed PCNA (this study), *Homo sapiens* (***H.s.***) ATAD5 (AlphaFold prediction AF-Q96QE3-F1), ***S.c.*** RFC–closed PCNA-DNA [Protein Data Bank (PDB) ID 7TID], ***S.c.*** Rad24-RFC–closed 9-1-1 clamp–DNA (PDB ID 7SGZ), and ***S.c.*** Ctf18 (AlphaFold prediction AF-P49956-F1). The PIP motifs are boxed by red dashed box, and the consensus feature is shown at the bottom. (**D**) An enlarged view at the interface of Elg1 and PCNA-1. The PCNA is shown as a transparency surface colored by the local hydrophobicity (left) and cartoon view in ivory (right). Interacting residues are in sticks and labeled (black for PCNA) and green for Elg1 residues. H-bonds are shown as cyan dashed lines.

In the structure of Elg1-RFC bound to a closed PCNA ring, the first three subunits—Elg1, Rfc4, and Rfc3—interact with PCNA (Fig. 5A). In comparison, in the structure of RFC bound to a closed PCNA ring that encircles DNA, the first four subunits—Rfc1, Rfc4, Rfc3, and Rfc2—interact with PCNA (Fig. 5B). An analysis of the buried solvent-accessible surface area showed that the Elg1-RFC–PCNA complex has a total buried surface of 1558 Å^2^, which is 56% smaller than the 2800 Å^2^ buried surface in the RFC-PCNA-DNA complex structure. In the Elg1-RFC–PCNA complex, Elg1 contributes about half of the binding interface (760 Å^2^ of 1558 Å^2^), while, in the RFC-PCNA-DNA complex, Rfc1 and Rfc3 each contribute one-third of the total interface with PCNA (955 and 1029 Å^2^, respectively, of 2800 Å^2^). Therefore, the Elg1-RFC unloader likely binds weaker than RFC to PCNA, indicating a more transient PCNA unloading process by Elg1-RFC compared to PCNA loading by RFC. This is consistent with our observation that a chemical cross-linker was needed to stabilize the Elg1-RFC–PCNA complex for visualization by cryo-EM, whereas no cross-linker was required to capture the RFC-PCNA-DNA complex (*8*), and is further supported by the larger angle (44°) between the Elg1-RFC unloader and PCNA ring compared to the angles between the RFC loader and PCNA with or without DNA (25° and 38°, respectively) (fig. S5).

PCNA binding proteins typically interact with the PCNA ring through a PCNA-interacting peptide (PIP) motif by inserting the peptide into two hydrophobic pockets on the PCNA surface, which follows the widely defined canonical feature: QxxΨxxΦΦ (x, any residue; Ψ, hydrophobic; and Φ, aromatic), while, for the noncanonical PIP motifs, the underlined positions can be varied (*50*). By a structural-based sequence alignment, we identified an eight-residue segment in Elg1 (^383^DFTTTHYV^390^) with the characteristics that fits a noncanonical PIP motif (Fig. 5C). Therefore, Elg1 interacts with PCNA using the general pattern of many other PCNA-binding proteins. Close examination of the binding interface shows that Elg1 binds the PCNA-1 protomer via a mixture of hydrophobic and hydrophilic interactions (Fig. 5D). The Elg1 residues Asp^383^, Thr^387^, Tyr^389^, and Val^390^ insert into the two small hydrophobic pockets of PCNA, and the Elg1 residues His^388^ and Arg^431^ sandwich and form hydrophobic stacking with the PCNA Phe^254^ (Fig. 5D, left). The Elg1-PCNA interaction further involves three H-bonds: the Elg1 Gln^362^, Thr^386^, and His^388^ with PCNA Arg^44^, Phe^254^, and Pro^252^, respectively (Fig. 5D, right).

### Characterization of Elg1-RFC PCNA unloading from DNA

#### Elg1-RFC only requires ATP binding for PCNA unloading

**Fig. 6.**
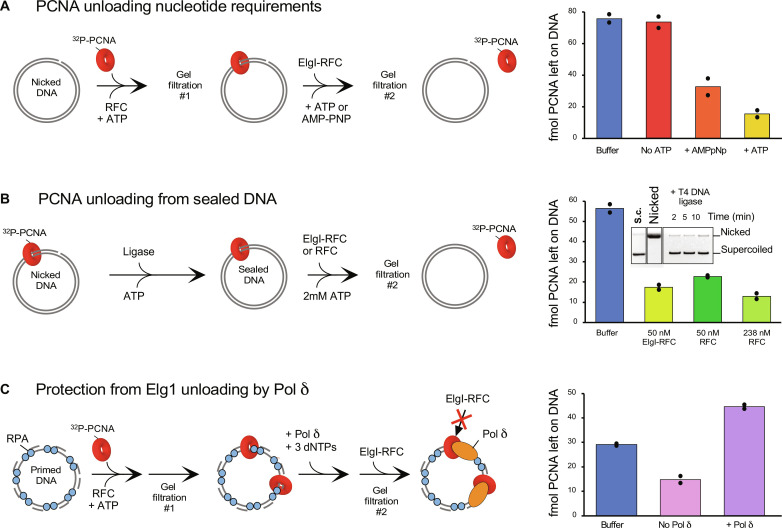
Characterization of Elg1-RFC unloading activity. (**A**) Binding of nucleotide is sufficient for Elg1-RFC to unload PCNA from DNA. ^32^P-PCNA is first loaded onto nicked plasmid using RFC and then gel-filtered to remove unbound ^32^P-PCNA (scheme). Elg1-RFC is added for 2 min with either buffer Elg1-RFC, Elg1-RFC, and ATP, or Elg1-RFC and AMP-PNP. The reaction is then gel-filtered a second time to quantify PCNA remaining on DNA (i.e., fractions 10 to 15), shown as a bar plot to the right. (**B**) Both RFC and Elg1-RFC can unload PCNA from a covalently closed duplex plasmid. After the first gel filtration, the DNA is sealed with ligase then treated with either RFC or Elg1-RFC followed by gel filtration (scheme). Quantification of PCNA remaining on DNA (i.e., fractions 10 to 15) is shown as a bar plot to the right. The inset within the bar plot is a native agarose gel showing that ligase sealed nicked DNA within 2 min. (**C**) Pol δ protects PCNA from being unloaded by Elg1-RFC. ^32^P-PCNA was loaded onto multi-primed M13mp18 ssDNA coated with RPA and then gel-filtered. Pol δ when then added (or not) for 2 min, followed by Elg1-RFC and ATP for 2 min. Quantification of PCNA remaining on DNA (i.e., fractions 10 to 15) is shown as a bar plot to the right. Some PCNA spontaneously dissociated during the 4-min reaction, as seen in the buffer control, but Pol δ stabilized PCNA on DNA even in the presence of Elg1-RFC. For these gel filtration experiments, duplicate experiments were performed on different days, the individual data points are shown in the histogram, and the primary data are shown in table S3.

Given that Elg1-RFC is not able to load PCNA, we wished to determine whether ATP hydrolysis or only ATP binding was required for PCNA unloading. To test this, we first isolated ^32^P-PCNA loaded onto a nicked plasmid as in the experiments of Fig. 1, and, then, the ^32^P-PCNA-DNA complex was treated for 2 min with either buffer alone, Elg1-RFC alone, or Elg1-RFC with either 2 mM ATP or 2 mM non-hydrolyzable AMP-PNP (scheme in Fig. 6A). The results show that Elg1-RFC can unload PCNA from DNA with non-hydrolyzable AMP-PNP (Fig. 6A). This result is consistent with a study using the weakly hydrolyzable ATPγS (*41*). Structure-based sequence alignment in the Walker A/P-loop and Walker B/DEAD box motif region showed that both Elg1 motifs vary somewhat relative to the five RFC subunits Rfc1 to Rfc5, and, unexpectedly, the Elg1 Walker A motif is one residue shorter (figs. S4 and S7, A and B). Furthermore, the Elg1 ATP binding site lacked the expected density for Mg^2+^ ion in the Elg1-RFC EM maps (fig. S7C). Together, these unique features suggest that ATP may not be hydrolyzed in the Elg1 ATP binding site.

#### Both Elg1 and RFC can unload PCNA from a sealed DNA

Previous work showed that RFC encircles the ss/dsDNA junction in the inner chamber of the clamp loader during PCNA loading (*6*, *49*, *51*). RFC binding to 3′ ss/dsDNA requires a sharp bend in the DNA, thought to specify loading to a nick or gap and not enabling loading on closed dsDNA (*52*). This action is essential to position DNA through the clamp and to specify its location to a replication terminus. In this reaction, RFC is directly aligned with PCNA for intimate connection to one face of PCNA needed to open and close the clamp. Thus, one might assume that RFC unloading of PCNA may involve the same initial steps that allow RFC to encircle ds/ssDNA and fully join with PCNA for clamp opening. Specifically, the binding of a primed ss/ds junction to the inner chamber of RFC would enable RFC to engage the full PCNA ring and open it for unloading, as it does during clamp loading. This conjecture follows the principle of microscopic reversibility. Thus, if this principle is correct for RFC loading/unloading, then it predicts that RFC will not unload PCNA from a sealed dsDNA circle (e.g., nick sealed by ligase) because it would not have interruptions in the DNA backbone to provide the requisite flexibility to encircle an ss/dsDNA junction in the central chamber.

The structure of Elg1-RFC indicates that it cannot bind dsDNA in the central chamber or the shoulder binding site. This strongly implies that PCNA unloading by Elg1-RFC does not require encirclement of dsDNA by the Elg1-RFC clamp unloader. It is suggested that Elg1-RFC only functions to remove PCNA from DNA after Okazaki fragments have been ligated (*39*), a structure that RFC may not be able to unload PCNA because the ss/dsDNA junction at the nick would be resealed and no longer flexible.

To test whether Elg1-RFC and/or RFC can unload PCNA from sealed dsDNA, we sealed the nicked DNA plasmid to form relaxed closed circular DNA and then examined unloading ability of Elg1-RFC and RFC. Using the same amount of Elg1-RFC or RFC, the Elg1-RFC1 appears only slightly superior to RFC (Fig. 6B). Use of four times the amount of RFC compared to that of Elg1-RFC was required to observe similar PCNA unloading as Elg1-RFC (Fig. 6B). The fact that RFC can remove PCNA from sealed DNA indicates that unloading is not on the reaction path of PCNA loading, given that RFC requires an ss/dsDNA junction for loading. This, in turn, suggests that Elg1-RFC may unload PCNA without requiring it to bind DNA in the central chamber.

#### Pol δ protects PCNA unloading from Elg1-RFC

Considering that Elg1-RFC binds the same interface of PCNA as Pol δ, it seems likely that Pol δ will prevent PCNA from unloading while idling as a Pol δ–PCNA complex on a primed site. To test this, we primed a ϕX174 ssDNA circle with 18 approximately equidistant 30-mer primers and coated it with RPA. We then loaded ^32^P-PCNA using RFC and gel-filtered the reaction to remove unbound ^32^P-PCNA (scheme in Fig. 6C). Elg1-RFC removed most of the ^32^P-PCNA from the primed DNA (Fig. 6C). In a parallel reaction, the ^32^P-PCNA–primed ssDNA was incubated with Pol δ in the presence of two deoxynucleoside triphosphates and treated with Elg1-RFC. The results showed full retention of ^32^P-PCNA on the DNA, revealing that Pol δ prevents Elg1-RFC unloading action, as expected. A control, adding no Pol δ or Elg1-RFC, showed some ^32^P-PCNA spontaneously dissociated from the primed RPA-coated ssDNA, indicating that PCNA is less stable on this template compared to a nicked duplex DNA. Thus, we can also conclude that Pol δ stabilizes the PCNA at a primed site. Overall, the results of these biochemical experiments confirm and extend a previous report on the eukaryotic ATAD5-RFC clamp unloader (*24*, *41*, *43*).

## DISCUSSION

### Elg1 structural elements that prevent loading of PCNA

The structures shown in this report clearly explain why Elg1-RFC is unable to load PCNA onto DNA. First, a large “plug” fills the inner DNA binding chamber, preventing DNA occupancy. Second, there are two locking loops of Elg1-RFC that prevent the ATP binding–dependent conformation changes required of RFC and Rad24-RFC to bind DNA for loading their respective clamps (i.e., PCNA and 9-1-1, respectively).

### Elg1-RFC–mediated PCNA unloading from different DNA structures

**Fig. 7.**
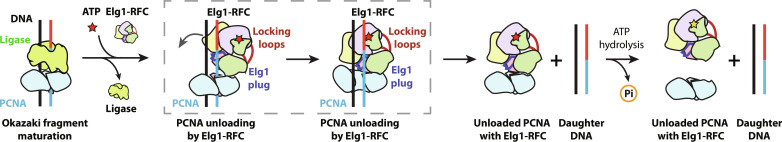
Hypothetical model of PCNA unloading by Elg1-RFC. After maturation of an Okazaki fragment, i.e., nick is sealed by DNA ligase, as previously proposed (*53*, *54*), the ligase will dissociate from PCNA to enable Elg1-RFC access to PCNA and unload it from DNA. We propose the Elg1-RFC initially approaches PCNA encircling the dsDNA at a large angle, with the Elg1 subunit first binding PCNA. The unloader then gradually tilts downward (shown by the black curved arrow) to further engage the PCNA. The binding energy likely cracks open the PCNA ring (see also fig. S5 for more details). Because of the presence of the Elg1 locking loops (in the shoulder) and Elg1 plug (in the central chamber), the approaching Elg1-RFC will push the daughter DNA out of the PCNA ring through the open PCNA gate, leading to PCNA unloading and dissociation of Elg1-RFC–PCNA from DNA. Subsequent ATP hydrolysis by Elg1-RFC will lead to separation of Elg1-RFC from the unloaded PCNA, completing the unloading process. Note that the two steps in the dashed box have not been experimentally visualized, probably due to their highly transient nature.

PCNA unloading by Elg1-RFC during genome replication has been shown to be dependent on Okazaki fragment ligation because deletion of the ligase Cdc9 gene resembles the phenotype of an Elg1 deletion, leading to PCNA accumulation on chromatin (*39*). These earlier results implied that Elg1-RFC requires closed DNA to unload PCNA from DNA. However, we show here that Elg1-RFC can unload PCNA from nicked, gapped. and sealed DNA. On the basis of the observations made here and our structures of the Elg1-RFC and its complex with PCNA, we propose that the ligase has reduced affinity to the DNA in which the nick between two adjacent Okazaki fragments has been sealed and that the ligase no longer binds the dsDNA well and can easily be competed off the PCNA-DNA by Elg1-RFC (Fig. 7, left) (*53*, *54*).

### PCNA protection by Pol δ from unloading by Elg1-RFC

We previously showed that the *E. coli* Pol III replicase protected the *E. coli* β clamp from unloading by the *E. coli* γ complex unloader (*55*). Like Pol δ and Elg1-RFC, both the *E. coli* Pol and clamp unloader bind and thus compete for the same face of their clamp. We show in the current report that Pol δ protects the PCNA ring against Elg1 unloading, similar to observations in the *E. coli* system. It seems likely that most proteins that bind to PCNA will prevent unloading, as they would compete with Elg1-RFC for the same surface of PCNA, and this is also consistent with earlier studies of Elg1-RFC (*41*). One might reasonably assume that Elg1-RFC would not be able to remove PCNA from DNA, while PCNA was functioning with another binding partner.

### Different paths for PCNA unloading and PCNA loading

The established mechanism of PCNA loading involves several defined steps: (i) RFC binds PCNA; (ii) when RFC is loaded with ATP, it opens PCNA; (iii) 3′ ss/dsDNA enters the central chamber of RFC-PCNA via the opened clamp interface that is positioned immediately underneath a side opening in the clamp loader pentamer (e.g., between Rfc1/Rfc5) followed by (iv) the closing of PCNA clamp and (v) ATP hydrolysis and clamp loader ejection from the clamp-DNA complex.

Clamp loader–DNA structures, including RFC-PCNA, revealed that DNA makes a very sharp bend and is the explanation as to why clamp loaders are specific to ss/dsDNA junctions, because dsDNA does not have the ability to make the sharp bend [i.e., the bend observed in structure analysis of DNA–clamp loader–clamp complexes (*56*)]. Thus, if clamp unloading were on the pathway of clamp loading, then the DNA that clamps are unloaded from would need to bind the central chamber of RFC and to also have the flexibility of an ss/dsDNA junction to bend out the side of the clamp unloader. The finding that RFC, the consensus clamp loader, can unload PCNA from a closed plasmid (this report) indicates that RFC unloading from dsDNA is likely different from the currently envisioned clamp loading mechanism. Given that Elg1-RFC shares this attribute that implies they may share central aspects of the unloading mechanism, the details of which will require further study.

### Hypothetical model of PCNA unloading by Elg1-RFC

To unload a clamp, one only needs to destabilize an interface between clamp subunits; unlike clamp loading that requires (i) destabilizing an interface in the PCNA clamp, (ii) stabilization of a stably wide-open clamp, and (iii) positioning of DNA through the opened clamp so it can close around DNA. However, clamp unloading only requires the first step, destabilization of an interface in the clamp, and, possibly, no long-lived open clamp intermediate. For example, only one monomeric subunit of the *E. coli* clamp loader distorts the β clamp dimer interface and unloads it from DNA (*57*, *58*). This may not be unexpected given that entropy is on the side of clamp unloading, which produces one more product molecule than the loading reaction (e.g., a free clamp).

To unload PCNA, we propose, based on the structures shown here, that Elg1-RFC approaches the DNA with a large angle because DNA cannot enter the complex and thus possibly mimics the Elg1-RFC–closed PCNA structure (Fig. 7, middle). The larger approaching angle of Elg1-RFC than that of the RFC supports this suggestion (Fig. 5, A and B). Elg1 subunit–PCNA interaction might destabilize one PCNA interface, as observed for the delta subunit of the *E. coli* clamp–DNA complex, unloading the clamp from DNA (*57*, *58*). Once the interface of PCNA is cracked, it likely dissociates from DNA (Fig. 7, right). It is also possible that the Elg1-RFC AAA^+^ domains may interact more fully with a transient destabilized form of PCNA, leading to the cracked ring structure observed herein.

In overview, this study reveals why Elg1-RFC is prevented from loading PCNA onto DNA. PCNA loading is clearly blocked by two loops of Elg1 that prevent the necessary conformation changes needed for DNA binding, as well as by an “Elg1 plug” that fills the central DNA binding chamber used to load PCNA onto DNA by RFC (Fig. 2B). Thus, after binding of Elg1-RFC to PCNA and PCNA ring cracking, the PCNA ring can only be unloaded from DNA and not loaded onto DNA. Therefore, given the great uncertainty about the clamp loading versus clamp unloading activities in the literature (*15*, *44*), the present study provides structural explanation why the Elg1-RFC is exclusively a PCNA unloader and distinguishes this alternative RFC complex from those that are primarily clamp loaders.

Further efforts are needed to understand how PCNA is taken off DNA by the unloader, perhaps by biochemically stabilizing an unloading intermediate complex in which Elg1-RFC binds to the PCNA while encircling a dsDNA substrate and then visualizing the intermediate structure by cryo-EM analysis.

## MATERIALS AND METHODS

### Reagents and proteins

Radioactive nucleotides were from PerkinElmer Life Sciences (Waltham, MA). Unlabeled ATP was from Cytiva (Marlborough, MA). ATPγS and AMP-PNP were from Roche (Basel, Switzerland). Apyrase from potato was from Sigma-Aldrich (St. Louis, MO). DNA-modifying enzymes were from New England Biolabs (NEB; Ipswich, MA). Protein concentrations were determined with the Bradford Protein stain (Bio-Rad Labs, Hercules, CA) using bovine serum albumin (BSA) as a standard. PCNA containing a hexahistidine tag and a six-residue site for the catalytic subunit of cyclic adenosine 3′,5′-monophosphate (cAMP)–dependent protein kinase A at the N terminus was cloned, expressed, purified, and radiolabeled with ^32^P-ATP as described earlier (*8*, *59*). For ^32^P-PCNA loading, we used the yeast RFC lacking the N-terminal residues 3 to 273 as described (*60*). Full-length RFC was used for ^32^P-PCNA unloading experiments (*61*). RPA was purified as described (*62*). S.c. Pol d was purified as described (*63*). ϕX174 ssDNA was from Roche (Basel, Switzerland). The 18 30-mer DNA oligos that hybridize nearly equally around the ssDNA of ϕX174 ssDNA were from IDT (see table S2).

### Plasmid DNAs with a single nick or a 20-nt gap and multi-primed ϕX174 ssDNA

Singly nicked pUC19 plasmid DNA was made using Nb. Bsm I that makes an ssDNA nick at a single site on the plasmid. To make the 20-nt ssDNA gap, a sequence that makes use of three unique ssDNA restriction enzymes was cloned into the Pci I and Bsp QI (NEB) sites of pUC19. To make this plasmid, pUC19 was digested with both Pci I and Bsp QI and the large DNA fragment was gel-purified from a native agarose gel. Then, oligos (5′-CATGTCCTCAGCAAGGAATGCAGAACC-3′ and 5′-GCTGGTTCTGCATTCCTTGCTGAGGA-3′ (Integrated DNA Technologies, Coralville, IA) were annealed in hybridization buffer [50 mM tris-HCl (pH 8.4), 1.5 M NaCl, and 1 M Na-citrate] by heating to 95°C in a heat block for 2 min and then allowed to cool to 23°C. The digested pUC19 and the annealed linkers were then ligated overnight at 15°C with T4 DNA ligase (NEB) at a vector:insert molar ratio 1:100. The ligation reaction was then transformed into DH5α competent cells (Invitrogen, Waltham, MA) and plated on LB and ampicillin (100 μg/ml). Minipreps from colonies were screened by sequencing over the inserted region before making a large-scale recombinant plasmid prep, referred to below as pNN1.

To make the 20-nt ssDNA gap, 92 μg of pNN1, in 2 ml of 50 mM tris-HCl (pH 7.9), 100 mM NaCl, 10 mM MgCl_2_, and BSA (100 mg/ml), was nicked with 200 U each of Nb. Bsm I, Nb. Bbv CI, and Nt. Bsp QI for 1.5 hours using 30 min for each enzyme at 65°, 37°, and 50°C, respectively. EDTA was then added to 25 mM, and the enzymes were heat-inactivated at 80°C for 30 min. The result is three nicks producing two adjacent 10-nt segments. To ensure complete removal of the 10-nt segments, a 100-fold molar excess of 5′-CAGAACCAGC-3′ and 5′-GCAAGGAATG-3′ (primers complementary to the two 10-nt segments generated by the nicking reaction) was added followed by heating the reaction to 55°C and then cooled slowly to room temperature. The reaction was then purified using Macherey-Nagel NucleoSpin Gel and PCR Clean-up columns (Takara Bio, USA, ref no. 740609.10) eluted in 5 mM tris-HCl (pH 8.5) and 1 mM EDTA. While the resulting plasmid migrated similar to a nicked DNA (i.e., not supercoiled) in a native agarose gel, we confirmed the presence of a 20-nt gap in the plasmid by adding a complementary 20-nt oligo and ligation with T4 DNA ligase. The result showed that >80% of plasmid treated in this way migrated as a supercoiled plasmid DNA, while reactions lacking the 20 mer did not change mobility in an agarose gel, confirming the majority of triple-nicked plasmid DNA contained the 20-nt gap.

Multi-primed ϕX174 ssDNA was made by using 18 30-mer oligonucleotide primers (sequences shown in table S2) that were annealed to the complementary ϕX174 virion ss circular DNA in an evenly spaced manner. S.c. RPA (454.5 nM) was then incubated with the multi-primed ϕX174 ssDNA at 30°C for 5 min.

### Elg1-RFC purification

The five different subunits encoding S.c. Elg1-RFC were expressed in *E. coli* using two expression plasmids having different and compatible origins as described previously (*61*). Genes encoding 3×FLAG-Elg1 and Rfc5 were cloned behind T7 RNA polymerase promotors in the pLANT-2RIL plasmid and co-transformed with pET(11a)-Rfc[2+3+4] into BLR(DE3) cells (Novagen, Madison, WI). Fresh transformants were grown in 12 liters of LB medium containing ampicillin (100 μg/ml) and kanamycin (50 μg/ml) at 37°C until the cell culture reached an optical density at 600 nm value of 0.6. Cell cultures were brought to 15°C by swirling in an ice water bath and then placed into a prechilled shaker incubator at 15°C. Protein expression was then induced upon adding 1 mM isopropyl-β-d-thiogalactopyranoside followed by 10 hours of incubation at 15°C. Induced cells were collected by centrifugation, and 2 mM PMSF was added and then lysed by two passages through an Avestin Emulsi Flex-C-50 (Ottawa, ON, Canada) at 4°C. Cell debris was removed by centrifugation at 19,000 rpm in an SS-34 rotor for 1 hour at 4°C. Cell lysate was incubated with 2 ml of anti-FLAG resin (MilliporeSigma, Burlington, MA) at 4°C for 1 hour and then pelleted at 1250 rpm at 4°C. The supernatant was discarded, and beads were washed three times by resuspension in 10 ml of buffer A [20 mM tris-HCl (pH 7.5), 1 M NaCl, 1 mM dithiothreitol (DTT), 1 mM EDTA, 1 mM PMSF, and 10% glycerol] followed by centrifugation at 1250 rpm at 4°C. The washed beads were resuspended in 10 ml of buffer A and poured into a 1-ml fast protein liquid chromatography column (Cytiva, Marlborough, MA) and then washed overnight with 20 ml of buffer A. Elution used 2 ml of pulses of 3×FLAG peptide (0.2 mg/ml) in buffer A for 30 min. Elg1-RFC appeared >90% pure by 10% polyacrylamide gel electrophoresis and was further purified on a 1-ml MonoS column (Cytiva, Marlborough, MA) using a gradient of 100 to 500 mM NaCl in 20 mM tris-acetate (pH 6.5), 1 mM DTT, 0.2 mM EDTA, 1 mM Mg-acetate, and 10% glycerol. For cryo-EM, the glycerol was removed by using a 5-ml ZEBA column (Thermo Fisher Scientific, Waltham, MA); equilibrated in 20 mM tris-acetate (pH 7.5), 1 mM DTT, 0.2 mM EDTA, 250 mM NaCl, and 1 mM Mg-acetate; and then rapidly concentrated using an Amicon Ultra 0.5 cell, 10K MWCO (EMD Millipore, Burlington, MA). A 10% polyacrylamide gel of the Elg1-RFC preparation is shown in fig. S1A. Elg1-RFC was aliquoted, flash-frozen in liquid N_2_, and stored at −80°C.

### ^32^P-PCNA loading and unloading assays by RFC and Elg1-RFC

#### PCNA loading reactions

The S.c. PCNA that was cloned with a six-residue N-terminal kinase recognition motif was radiolabeled with γ-[^32^P] ATP using the recombinant catalytic subunit of cAMP-dependent protein kinase as described (*59*, *64*). The radiolabeled PCNA was then assembled onto a singly nicked plasmid or a multi-primed ϕX174 ssDNA template in a reaction containing 3 nM ^32^P-PCNA (128 cpm/fmol), 5.9 nM Nt. Bsp QI–nicked pUC19 (or 8 nM 18-primer–ϕX174 template precoated with 454.5 nM S.c. RPA), 11.5 nM nM RFC, and 2 mM ATP in 434 μl of buffer B [20 mM tris-HCl (pH 7.5), 0.5 mM EDTA, BSA (0.1 mg/ml), 4% glycerol, 8 mM MgCl_2_, and 2 mM DTT] with 50 mM NaCl. After incubating the mixture for 15 min at 30°C, 11.4 U of apyrase was added and incubated for another 5 min to destroy any remaining ATP (confirmed by TLC). Then ^32^P-PCNA-DNA complex was purified from free ^32^P-PCNA by gel filtration on 5-ml Bio-Gel A15m columns (Bio-Rad Laboratories, Hercules, CA) equilibrated in buffer B containing 100 mM NaCl. Fractions of 180 μl were collected and counted by liquid scintillation. The molar amount of ^32^P-PCNA in each fraction was calculated from the known specific radioactivity of PCNA determined empirically on the day of the experiment. This “isolated PCNA-DNA” complex was aliquoted and frozen at −80°C for the remaining experiments.

#### Elg1-RFC activity in unloading PCNA from nicked DNA

Reactions were examined for ^32^P-PCNA clamp unloading using isolated ^32^P-PCNA-DNA complex as described above, performing the reactions and pooling the peak ^32^P-PCNA-DNA-bound fractions (nos. 11 to 16). Unless indicated differently, unloading reactions contained 0.69 nM ^32^P-PCNA-DNA, 56 nM Elg1-RFC (or RFC), and 2 mM ATP (or AMP-PNP) and then incubated in 200 μl of buffer B with 100 mM NaCl for 2 min at 30°C before gel filtration on a 5-ml Bio-Gel A15m column equilibrated in buffer B containing 100 mM NaCl. Fractions of 180 μl were collected and counted by liquid scintillation. The molar amount of ^32^P-PCNA in each fraction was calculated from the known specific radioactivity of ^32^P-PCNA.

#### Elg1-RFC activity in unloading PCNA from sealed DNA

To examine PCNA unloading by Elg1-RFC (or RFC) from a sealed DNA plasmid, the nick on the “isolated ^32^P-PCNA-DNA” was sealed with ligase in a reaction that contained 0.69 nM ^32^P-PCNA–nicked DNA complex, 1 mM ATP, and 8000 U of T4 DNA ligase in 203 μl of buffer B with 100 mM NaCl and was incubated at 30°C for 3 min (confirmed by thin-layer chromatography). The PCNA-unloading reaction was then initiated upon adding 52 nM Elg1-RFC (or RFC) and 1 mM ATP to a final volume of 215 μl. The reaction was incubated for 2 min at 30°C before gel filtration over a second 5-ml BioGel A15 column using buffer B with 100 mM NaCl. Fractions were collected and radioactivity counted by liquid scintillation as described above.

#### Pol δ protection from Elg1-RFC PCNA unloading activity

To determine whether Pol δ could protect the PCNA on DNA from being unloaded by Elg1-RFC, 0.55 nM ^32^P-PCNA-RPA–coated multi-primed ϕX174 DNA complex was first incubated with 117 nM S.c. Pol δ in 207 μl of buffer B containing 100 mM NaCl; 1.9 mM ATP; and 0.48 mM dATP, dGTP, and dTTP. This Pol δ idling reaction was incubated for 2 min at 30°C before 80.28 nM Elg1-RFC was added (final volume of 213 μl) and incubated for another 2 min. The reaction was quenched with 22.4 mM EDTA and then applied to a second 5-ml BioGel A15 column using buffer B with 100 mM NaCl. Fractions were collected and radioactivity counted by liquid scintillation as described in the previous paragraph.

### In vitro assembly of the Elg1-RFC–PCNA complex in the presence of DNA

The assembly of yeast Elg1-RFC, PCNA clamp, and DNA complex followed our previous reports (*8*, *10*). The DNA substrate was synthesized and annealed by IDT Inc. and contained a 30-nt primer (5′-GCCTAGCTCGACGCCATTAATAATGTTTTC-3′) and a 50-nt template (5′-GAAAACATTATTA ATGGCGTCGAGCTAGGCACAAGGCGAACTGCTAACGG-3′) with a 30–base pair ds region. For in vitro reconstitution, 0.45 μl of purified PCNA clamp (43.8 μM) and 4.3 μl of DNA substrate (100 μM) were mixed and incubated at 30°C for 10 min with 0.75 μl of ATPγS (10 mM) and 1 μl of Mg-acetate (100 mM). This is followed by the addition of 8.5 μl of Elg1-RFC (5.1 μM). The final concentrations of these components in the 15-μl reaction volume were as follows: Elg1-RFC at 2.9 μM, PCNA clamp at 1.3 μM, DNA substrate at 28.7 μM, ATPγS at 0.5 mM, and Mg-acetate at 6.7 mM, equivalent to a final molar ratio of 1.0 (Elg1-RFC):0.4 (PCNA clamp):10 (DNA). The mixture was then incubated in an ice-water bath for 15 min. In such reaction product, only the Elg1-RFC particles were observed in EM images. Varying the assembly time (from 15 to 10, 5, and 1.5 min) or the temperature (from 0° to 30°C) did not lead to the assembly of the Elg1-RFC–PCNA complex, based on two-dimensional (2D) classification and class-averaged images. This is indicative of weak and transient binding between Elg1-RFC and PCNA. We therefore added 1.7 μl of glutaraldehyde to the reaction mixture and incubated it for another 15 min in the ice-water bath before adding 1 M tris-HCl (pH 7.5; final concentration of 40 mM) to quench the cross-linking reaction. This modified procedure led to the successful capture of the Elg1-RFC–PCNA complex (fig. S1, D and E).

### Cryo-EM grid preparation and data collection

Quantifoil Au R1.2/1.3 300 mesh EM grids were used in this study. The EM grids were glow discharged for 30 s in a Gatan Solarus, and, then, 3 μl of the reaction mixture was applied onto the freshly treated grids. Vitrification of samples on EM grids was carried out in a Vitrobot (Thermo Fisher Mark IV) with the following settings: blot time of 3.5 s, blot force of 3, wait time of 3 s, and blotting chamber temperature of 6°C with 95% relative humidity. The blotted EM grids were flash-frozen in liquid ethane cooled by liquid nitrogen. Cryo-EM data were collected on a 300-kV Titian Krios electron microscope (×105,000 magnification) controlled by SerialEM (*65*) in a multi-hole mode with multiple shots in each hole. The micrographs were recorded on a K3 direct electron detector (Gatan) operated in the super-resolution video mode, with the objective lens under-focus values ranging from 1.1 to 1.8 μm. During a 1.3-s exposure time for each micrograph, a total of 65 frames were recorded with a total dose of 60 *e*^−^/Å^2^. The calibrated image pixel size was 0.828 Å at the specimen level. Two datasets were collected in two Krios sessions (2 to 3 days each), leading to high-resolution EM maps of the targeted complexes (fig. S2).

### Image processing and 3D reconstruction

The data collection and image quality were monitored by the cryoSPARC Live v4.0.0 (*66*) installed in a local workstation. The image preprocessing including patch motion correction on bin x2 data, contrast transfer function (CTF) estimation and correction, blob particle picking (70- to 150-Å diameter), and particle extraction on bin x4 data. A total of ~30,000 raw micrographs were recorded in the two data collecting sessions (fig. S2). The extracted particle images were then subjected to two rounds of 2D classification, resulting in a selected dataset of ~2.1 and 1.6 million “good” particle images from the original two datasets. We also trained the automatic particle picking program Topaz (*67*) and used the trained model to pick up more particles. The Topaz picked dataset, which was also subjected to two rounds of 2D classifications, resulted in the larger dataset of ~2.7 and 2.1 million good particle images. We then used the reported “Build and Retrieve” method to retrieve any less frequently occurring particle views (*68*). The two particle datasets were combined by removing duplicates with 40% overlapping (52 Å) or larger than the preset particle diameter (~130 Å). This resulted in a merged dataset of ~3.5 and 2.7 million particle images from each session (fig. S2).

Ab initio 3D reconstruction in cryoSPARC with four pre-set classes yielded two good initial maps: one Elg1-RFC alone and one Elg1-RFC bound to PCNA. The remaining two classes were considered “junk maps” as they did not have well-defined structural features. The two good maps were used as 3D references for subsequent heterogeneous, nonuniform, and local refinement, which yielded a 3.6-Å EM map of the Elg1-RFC alone and a 3.8-Å EM map of Elg1-RFC bound to a closed-ring PCNA from the first session data. Similar processing of the second dataset yielded the EM maps of Elg1-RFC alone and Elg1-RFC bound to a closed-ring PCNA both at 3.5-Å resolution (fig. S2, top). Then, particles belonging to the same structure from the two datasets were combined, and their coordinates were converted using the PyEM program (*69*) into the Relion format (*70*) for further processing.

In parallel, the raw micrographs from the two Krios sessions were imported into Relion 4.0 (*70*) for motion correction by MotionCor2 (*71*) and CTF estimation and correction by CTFFIND4 (*72*). Then, the particle images were reextracted with the above-derived particle coordinates imported from cryoSPARC. Further 3D classification was performed in Relion with four 3D classes for each state. For the Elg1-RFC alone state, only one good 3D class was kept, but two conformations were obtained for the Elg1-RFC–PCNA complex, one with a closed PCNA ring and the other with a cracked PCNA (fig. S2, middle). After 3D autorefinement, CTF refinement, and Bayesian polishing, the particles from each class were imported back to cryoSPARC for the final nonuniform refinement, which resulted in three final EM maps: an EM map for Elg1-RFC at 3.20 Å, an EM map for the Elg1-RFC–closed PCNA ring at 3.25 Å, and an EM map for the Elg1-RFC–cracked PCNA ring at 3.67 Å (figs. S2, bottom, and S3).

### Model building, refinement, and validation

The previously reported structure of the yeast RFC-PCNA clamp complex (Protein Data Bank entry 1SXJ) was used as the starting model for atomic model building of the Rfc2-5 and PCNA in the three EM maps (*6*). De novo model building by ModelAngelo and AlphaFold prediction (AF-Q12050-F1) was used to build the starting Elg1 model (*68*, *73*), along with the de novo modeling program Map-to-Model wrapped in Phenix (*74*). The starting Elg1 model and the yeast RFC-PCNA model were fitted into the EM map of the Elg1-RFC–closed ring PCNA. Then, the Rfc1 subunit in RFC was replaced by the Elg1 model to generate a single coordinate file in UCSF Chimera (*75*). The starting model was then refined iteratively between the real space refinement in Phenix and the manual adjustment in Coot (*76*). For several regions with weaker EM densities including two PCNA protomers lining the DNA entry/exit gate and the Elg1 locking loops, conventional rigid-body docking was performed, which was followed by manual adjustment in Coot and refinement in Phenix. The atomic model of the Elg1-RFC–closed PCNA complex was refined to 3.2 Å. The model was validated by the MolProbity program embedded in Phenix (*77*). This model then served as the initial model for modeling building into the EM maps of the Elg1-RFC alone and the Elg1-RFC–cracked PCNA, followed by a similar model building and validation process as described above (table S1). Structure figures were prepared in ChimeraX (*78*) and organized in Adobe Illustrator (Adobe Inc., San Jose, CA).

### Statistical analysis

Three atomic models built according to the cryo-EM maps are refined in Phenix, and statistics are obtained from Phenix comprehensive validation and listed in table S1. The PCNA loading or unloading data points in the biochemical assays are obtained from two or three independent experiments. Individual data points are listed in table S3.
