# Supplementary material for: Structure of the PCNA unloader Elg1-RFC
Source: Sci Adv. 2024 Mar 1;10(9):eadl1739. doi: 10.1126/sciadv.adl1739 (PMC10906927; doi:10.1126/sciadv.adl1739)
Supplement: Supplementary file 1 — Figs. S1 to S7 Tables S1 to S3 Legends for movies S1 and S2 [file sciadv.adl1739_sm.pdf]

Science Advances
AAAS

Supplementary Materials for

Structure of the PCNA unloader Elg1-RFC

Fengwei Zheng *et al.*

Corresponding author: Huilin Li, huilin.li@vai.org; Michael E. O'Donnell, odonnel@rockefeller.edu

Sci. Adv. **10**, eadl1739 (2024)
DOI: 10.1126/sciadv.adl1739

The PDF file includes:

Figs. S1 to S7
Tables S1 to S3
Legends for movies S1 and S2

Other Supplementary Material for this manuscript includes the following:

Movies S1 and S2

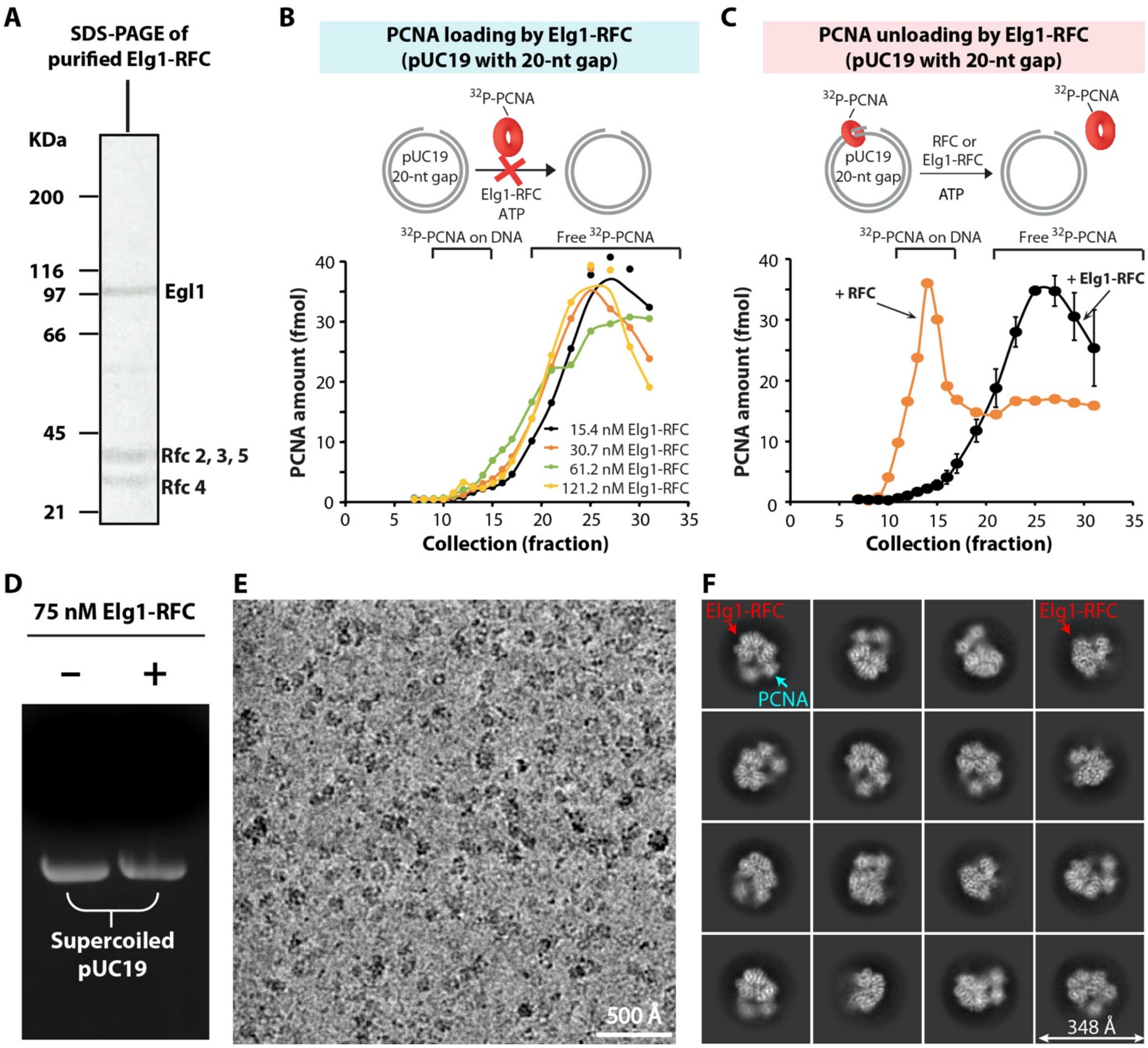

Fig. S1. Verification of *S. c.* Elg1-RFC activity and Elg1-RFC–PCNA complex assembly. (A), Elg1-RFC preparation in a 10% SDS-PAGE stained with Coomassie Blue. (**B**), Elg1-RFC at the indicated and elevated concentrations do not load the PCNA clamp onto a 20-nt gapped circular pUC19 plasmid, showing that possible ssDNA threading through the motors of Elg1-RFC does not lead to PCNA clamp loading. (**C**), Elg1-RFC can unload the PCNA clamp from the 20-nt gapped circular pUC19 plasmid. One experiment was performed for the buffer control, and three independent experiments were performed for Elg1-RFC in which data points are presented as the mean (filled circles) ± one standard deviation (error bars). See methods for details. (**D**), The Elg1-RFC preparations cannot cleave a supercoiled pUC19 plasmid, and thus results are not due to clamp loading and then sliding off from the end of linearized DNA. (**E**), A typical raw micrograph of the Elg1-RFC assembled with the PCNA clamp by directly mixing separately purified components *in vitro*. A total of 29,940 such micrographs were recorded in this study. (**F**), Selected 2D class averages in different views. The EM densities corresponding to Elg1-RFC and PCNA clamp are labelled in the first row of 2D averages. Scale bars are shown in the lower right panel.

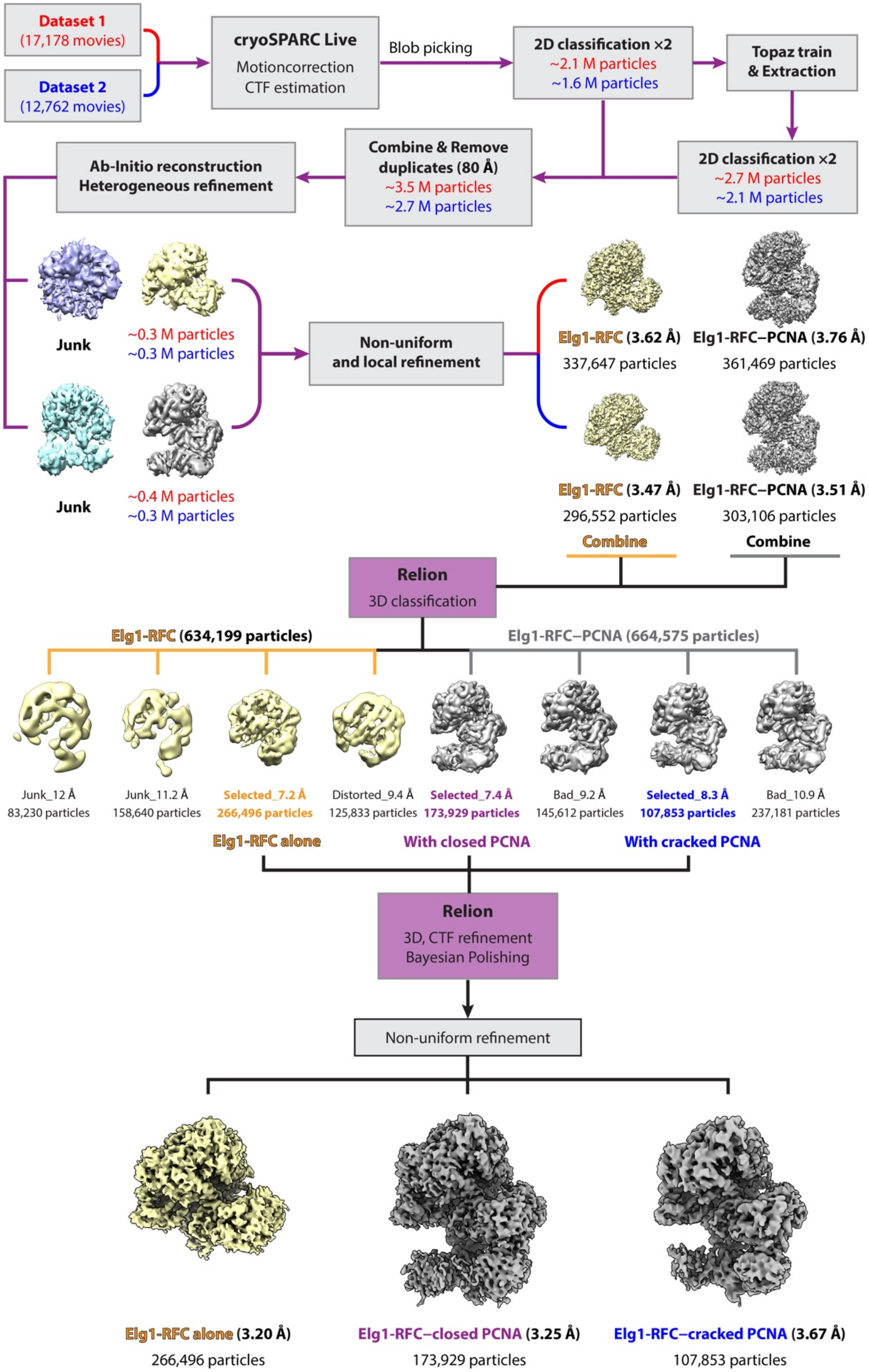

Dataset 1
(17,178 movies)
Dataset 2
(12,762 movies)
cryoSPARC Live
Motioncorrection
CTF estimation
Blob picking
2D classification ×2
~2.1 M particles
~1.6 M particles
Topaz train
& Extraction
2D classification ×2
~2.7 M particles
~2.1 M particles
Combine & Remove duplicates (80 Å)
~3.5 M particles
~2.7 M particles
Ab-Initio reconstruction
Heterogeneous refinement
Junk
~0.3 M particles
~0.3 M particles
Junk
~0.4 M particles
~0.3 M particles
Non-uniform and local refinement
Elg1-RFC (3.62 Å)
337,647 particles
Elg1-RFC–PCNA (3.76 Å)
361,469 particles
Elg1-RFC (3.47 Å)
296,552 particles
Elg1-RFC–PCNA (3.51 Å)
303,106 particles
Combine
Combine
Relion
3D classification
Elg1-RFC (634,199 particles)
Elg1-RFC–PCNA (664,575 particles)
Junk_12 Å
83,230 particles
Junk_11.2 Å
158,640 particles
Selected_7.2 Å
266,496 particles
Distorted_9.4 Å
125,833 particles
Selected_7.4 Å
173,929 particles
Bad_9.2 Å
145,612 particles
Selected_8.3 Å
107,853 particles
Bad_10.9 Å
237,181 particles
Elg1-RFC alone
With closed PCNA
With cracked PCNA
Relion
3D, CTF refinement
Bayesian Polishing
Non-uniform refinement
Elg1-RFC alone (3.20 Å)
266,496 particles
Elg1-RFC–closed PCNA (3.25 Å)
173,929 particles
Elg1-RFC–cracked PCNA (3.67 Å)
107,853 particles

Fig. S2. Workflow of cryo-EM data processing leading to one Elg1-RFC alone map and two Elg1-RFC-PCNA complex maps. Two datasets (1 and 2) with ~ 30,000 movies in total were collected in two Krios sessions, monitored by cryoSPARC Live (v4.0.0) with preprocessing. Further 3D reconstruction and refinement were performed in both cryoSPARC and Relion (v4.0 beta).

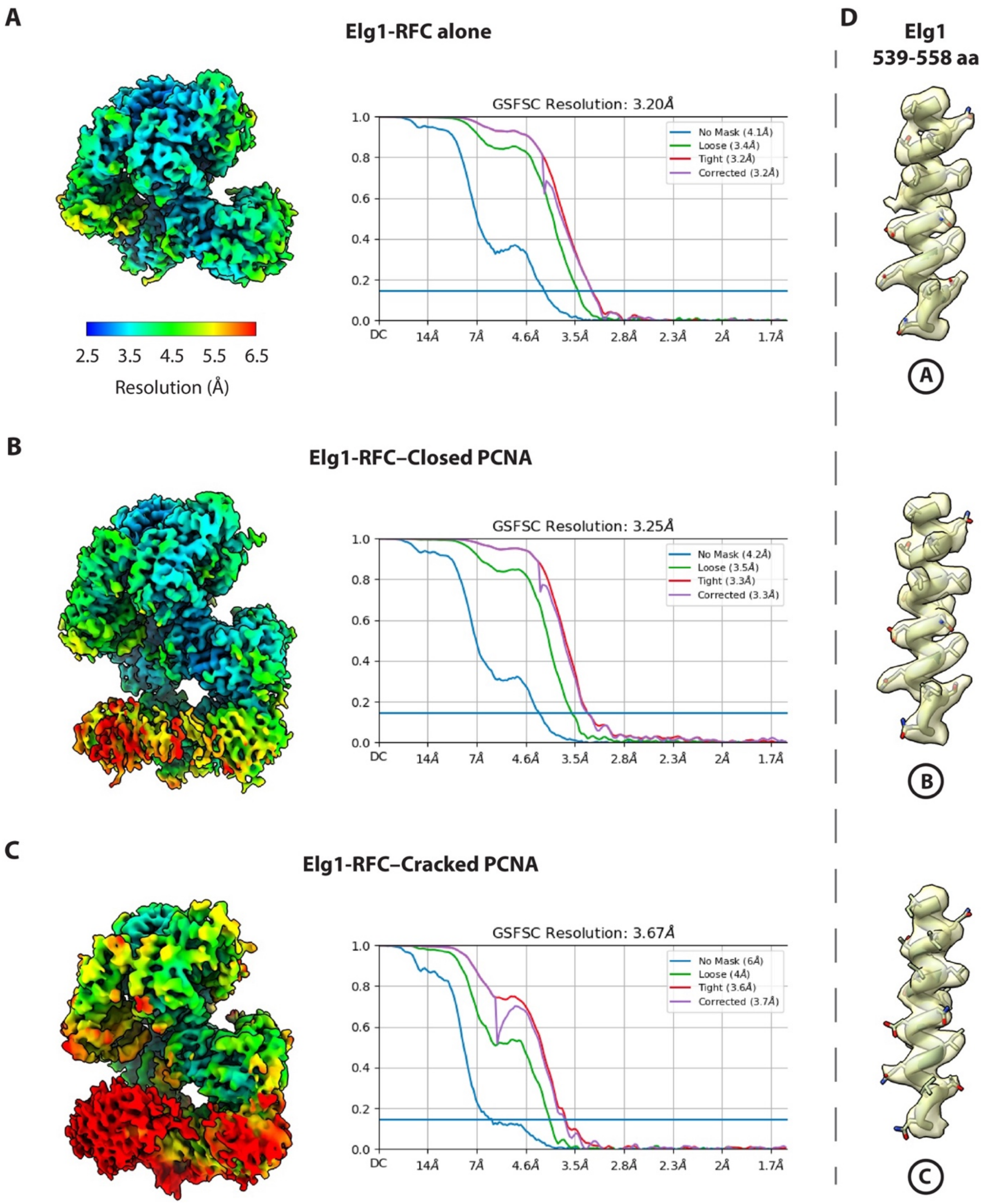

Fig. S3. Local resolution estimation of the three EM maps and the fit between map and model. **(A-C)**, Left: Color-coded local resolution map of Elg1-RFC alone (top), Elg1-RFC–closed ring PCNA (middle), and Elg1-RFC–cracked-ring PCNA (bottom). Right: Gold standard Fourier shell correlation curve (GSFSC) of the half-maps and the correlation curve of model and map of the corresponding complex. (**D**), The EM density map of a selected Elg1 α-helix (aa 539-558) superimposed with the atomic model from each of the three complexes.

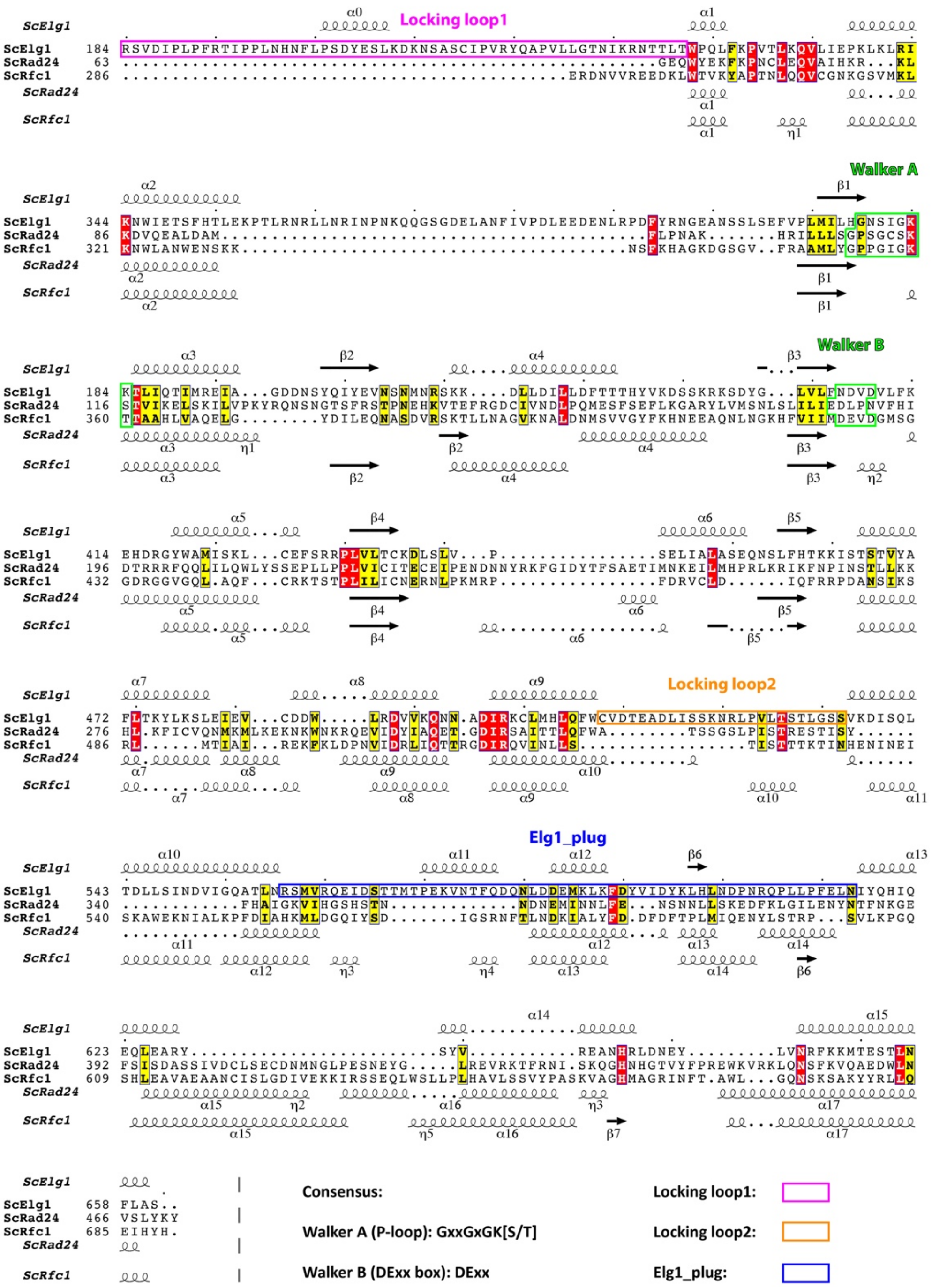

Fig. S4. Secondary structure-based sequence alignment of the *S. cerevisiae* Elg1, Rad24, and Rfc1. The subunit structures are available from these three complex structures: RFC (PDB entry 1SXJ), Rad24-RFC (PDB entry 7SGZ), and Elg1-RFC (this study). Note that only the structurally solved regions are included in the alignment. α-Helices and β-strands are shown above the sequences as coils and arrows, respectively. The assignments are produced by ESPript 3.0 (https://espript.ibcp.fr). Key features including the locking loops LL1 and LL2, Walker A and B, and the Elg1 plug) are marked above the sequences. Note that the Elg1 collar domain is unique containing the plug. See also main text for details and **Fig. 4**.

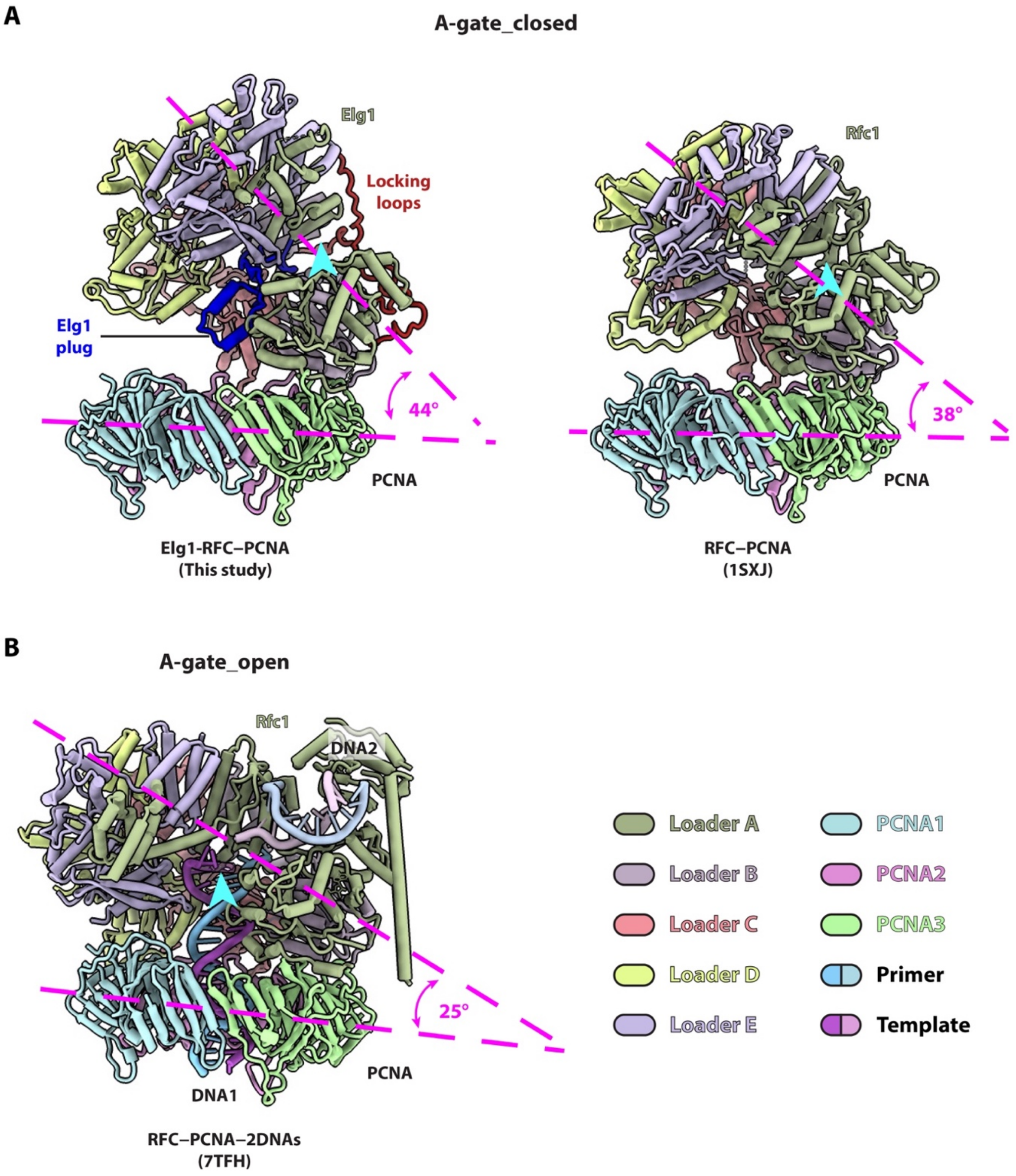

Fig. S5. Structural comparison of the PCNA loader RFC with the PCNA unloader Elg1-RFC. (**A)**, In the absence of DNA, both RFC and Elg1-RFC are in the A-gate closed state. The angle between Elg1-RFC and the PCNA clamp is about 6° larger than in RFC and PCNA (44° versus 38°, respectively). The larger angle may enable easier access of Elg1-RFC to dsDNA-encircling PCNA. (**B)**, In the presence of DNA, the RFC adopted an A-gate opened state. This ternary complex is more compact, as the RFC approaching angle is now narrowed to 25°. These angles were measured between a line running through the PCNA ring and another line crossing the interface between the Rfc5 AAA+ module and collar domain. The color scheme is shown at the bottom right. The A-gate in each structure is marked by a cyan arrow.

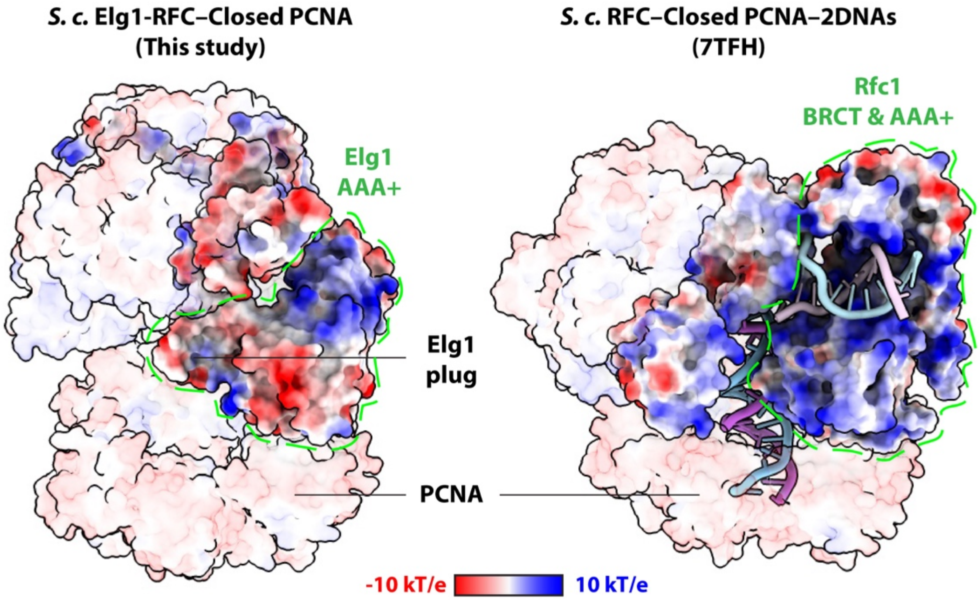

Fig. S6. Comparison of the surface electrostatic potential of Elg1 and Rfc1. The structures of Elg1-RFC–closed PCNA (this study) and 2 DNA molecules bound to RFC–closed PCNA (PDB entry 7TFH) are used for this comparison. Only Elg1 and Rfc1 are shown as surface charges, with the remainder of the structures shown as semi-transparent surface views. DNA molecules in RFC are shown as cartoons. Note that region encircled in the dashed green shape in Elg1-RFC encompassing the Elg1 AAA+ module is much less positively charged than the DNA-binding region in RFC encompassing the Rfc1 BRCT domain and AAA+ module.

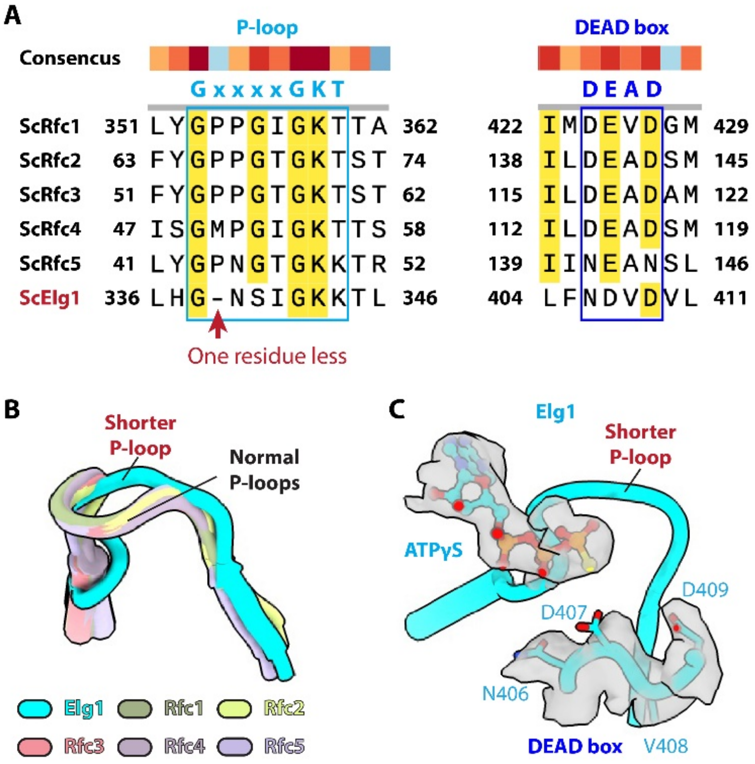

Fig. S7. The Elg1 ATP binding site is modified as compared with the five ATP binding sites in RFC. (A), Sequence alignment with the yeast RFC (PDB entry 7TFK) subunits Rfc 1 to 5. Both P-loop (**Left** panel) and DEAD box (**Right** panel) in Elg1 are varied compared with those of Rfc1-5. The Elg1 P-loop is one residue shorter. **(B)**, Structural alignment of the P-loops. The Elg1 P-loop (cyan) is one-residue shorter and in a distinct configuration compared with the five RFC subunits. **(C)**, There is no ATP-coordinated Mg^{2+} density in the Elg1 ATP binding pocket. For clarity, only the cryo-EM density within 3 Å around ATPγS and the DEAD box are shown (at the threshold level of 0.275).

Table S1. Cryo-EM data collection, refinement, and validation statistics

	#1 Apo Elg1-RFC (EMDB-41252) (PDB 8THB)	#2 Elg1-RFC–cracked PCNA (EMDB-41253) (PDB 8THC)	#3 Elg1-RFC–closed PCNA (EMDB-41254) (PDB 8THD)
**Data collection and processing**			
Magnification	105,000	105,000	105,000
Voltage (kV)	300	300	300
Electron exposure (e–/Å^2)	60	60	60
Defocus range (-μm)	1.1 – 1.8	1.1 – 1.8	1.1 – 1.8
Pixel size (Å)	0.828	0.828	0.828
Symmetry imposed	C1	C1	C1
Initial particle images (no.)	634,199	664,575	664,575
Final particle images (no.)	266,496	107,853	173,929
Map resolution (Å)	3.20	3.67	3.25
FSC threshold	0.143	0.143	0.143
Map resolution range (Å)	2.8 – 12.0	3.3 – 13.0	1.8 – 13.0
**Refinement**			
Initial model used (PDB entry)	#3 of this study	#3 of this study	1SXJ, AF-Q12050-F1
Model resolution (Å)	3.17	3.63	3.26
FSC threshold	0.143	0.143	0.143
Model resolution range (Å)	2.8 – 12.0	3.3 – 13.0	1.8 – 13.0
Map sharpening *B* factor (Å^2)	-115.3	-116.1	-94.3
Model composition			
Non-hydrogen atoms	14,334	20,430	20,396
Protein/DNA residues	1,782/0	2,558/0	2,553/0
Ligands	8	8	8
B factors (Å^2)			
Protein/DNA	92.71/0	129.78/0	86.04/0
Ligand	86.39	88.70	76.26
R.m.s. deviations			
Bond lengths (Å)	0.003	0.003	0.003
Bond angles (°)	0.549	0.637	0.652
Validation			
MolProbity score	1.54	1.61	1.66
Clashscore	8.27	8.26	8.30
Poor rotamers (%)	0.37	0.04	0.43
Ramachandran plot			
Favored (%)	97.56	97.12	96.72
Allowed (%)	2.44	2.88	3.28
Disallowed (%)	0	0	0

Table S2. Sequence of the 18 primers that were annealed to ϕX174 ssDNA to make the multi-primed DNA template used in PCNA unloading

1. AACTTCTGCGTCATGGAAGCGATAAAACTC
2. TAAAATGTCAACAAGAGAATCTCTACCATG
3. CGGCAGCAATAAACTCAACAGGAGCAGGAA
4. GCGCCTTTACGCTTGCCTTTAGTACCTCGC
5. ACGTGACGATGAGGGACATAAAAAGTAAAA
6. TCAGGAGGAAGCGGAGCAGTCCAAATGTTT
7. AAGAAACGCGGCACAGAATGTTTATAGGTC
8. GAAGGCGGTTCCTGAATGAATGGGAAGCCT
9. GTTTGAATTATGGCGAGAAATAAAAGTCTG
10. CAAAGGATAAACATCATAGGCAGTCGGGAG
11. GAATGCCACCGGAGGCGGCTTTTTGACCGC
12. CACGCTCCCAAGCATTAAGCTCAGGAAATG
13. TAAGCATTTGGCGCATAATCTCGGAAACCT
14. TCAATCCTGACGGTTATTTCCTAGACAAAT
15. GAACGTCAGAAGCAGCCTTATGGCCGTCAA
16. AACGCTGAATAGCAAAGCCTCTACGCGATT
17. GACCAAAATTAGGGTCAACGCTACCTGTAG
18. TTCTGAACAGCTTCTTGGGAAGTAGCGACA

Table S3. Individual quantitation of PCNA on DNA for each of the experiments of Figure 6.

Figure 6A data	exp 1 (fmol)	exp 2 (fmol)
buffer	74.41	77.36
no ATP	71.51	75.79
+ AMPpNp	29.20	36.08
+ ATP	18.83	16.66
Figure 6B data	exp 1	exp 2
buffer	55.19	57.60
+ 50 mM Elg1-RFC	17.01	17.65
+ 50 nM RFC	22.93	22.53
+ 238 nM RFC	13.56	12.45
Figure 6C data	exp 1	exp 2
buffer	29.17	28.98
no Pol delta	15.53	14.03
+ Pol delta	44.48	44.75

Supplementary Movie legends

Movie S1. A side-by-side comparison of the Elg1-RFC structure alone with the structures of Elg1-RFC bound to a closed-ring PCNA and to a partially cracked-ring PCNA. The three atomic models are rotated 360º around the Y axis, followed by a 360º rotation around the X axis. The models are aligned but shown separately.

Movie S2. A morph from Rfc1 to Elg1 to visualize how a loader is reconfigured into an unloader. The PCNA loader RFC (PDB entry 1SXJ) and the unloader Elg1-RFC (this study) used for morphing are both from yeast, in a similar state with a closed PCNA ring, a closed A-gate, and in the absence of DNA. Those two complexes are rotated 360º around the Y axis individually. Then the two structures are superimposed. All subunits except for Rfc1 and Elg1 fade away. Then the scene is zoomed in, starts to morph from Rfc1 to Elg1, and wobbles around the Y and X axes to visualize how the loader collar domain is rearranged into that of an unloader.
